# Rich RNA Structure Landscapes Revealed by Mutate-and-Map Analysis

**DOI:** 10.1371/journal.pcbi.1004473

**Published:** 2015-11-13

**Authors:** Pablo Cordero, Rhiju Das

**Affiliations:** 1 Biomedical Informatics Program, Stanford University, Stanford, California, United States of America; 2 Biochemistry Department, Stanford University, Stanford, California, United States of America; 3 Physics Department, Stanford University, Stanford, California, United States of America; University of Missouri, UNITED STATES

## Abstract

Landscapes exhibiting multiple secondary structures arise in natural RNA molecules that modulate gene expression, protein synthesis, and viral. We report herein that high-throughput chemical experiments can isolate an RNA’s multiple alternative secondary structures as they are stabilized by systematic mutagenesis (mutate-and-map, M^2^) and that a computational algorithm, REEFFIT, enables unbiased reconstruction of these states’ structures and populations. In an *in silico* benchmark on non-coding RNAs with complex landscapes, M^2^-REEFFIT recovers 95% of RNA helices present with at least 25% population while maintaining a low false discovery rate (10%) and conservative error estimates. In experimental benchmarks, M^2^-REEFFIT recovers the structure landscapes of a 35-nt MedLoop hairpin, a 110-nt 16S rRNA four-way junction with an excited state, a 25-nt bistable hairpin, and a 112-nt three-state adenine riboswitch with its expression platform, molecules whose characterization previously required expert mutational analysis and specialized NMR or chemical mapping experiments. With this validation, M^2^-REEFFIT enabled tests of whether artificial RNA sequences might exhibit complex landscapes in the absence of explicit design. An artificial flavin mononucleotide riboswitch and a randomly generated RNA sequence are found to interconvert between three or more states, including structures for which there was no design, but that could be stabilized through mutations. These results highlight the likely pervasiveness of rich landscapes with multiple secondary structures in both natural and artificial RNAs and demonstrate an automated chemical/computational route for their empirical characterization.

## Author Summary

RNA is a versatile macromolecule that underlies core natural processes throughout living systems and new strategies to re-engineer these systems. In several cases, this versatility is due to the ability of an RNA molecule to adopt multiple conformations: the full description of the molecule involves a ‘landscape’ of alternative structures that are present at equilibrium and whose interconversions are critical for function. However, there is little work on empirically probing these landscapes due to size, resolution, and infrastructure constraints of available experimental methods. We show herein that RNA landscapes can be characterized rapidly through the mutate-and-map (M^2^) methodology when coupled with a novel blind-source separation method, REEFFIT. We present extensive computational and experimental benchmarks supporting the use of M^2^-REEFFIT to detect alternative RNA secondary structures accurately without ‘over-predicting’ them. We then experimentally address a basic question in RNA biophysics—do landscapes involving multiple secondary structures require explicit selection or can they arise in artificial RNA sequences without selection? For both an artificial flavin mononucleotide riboswitch and a randomly generated sequence, M^2^-REEFFIT and further mutation tests find evidence for alternative, unexpected states, suggesting that rich landscapes are common and will become better appreciated with use of the developed technology.

## Introduction

**Fig 1:**
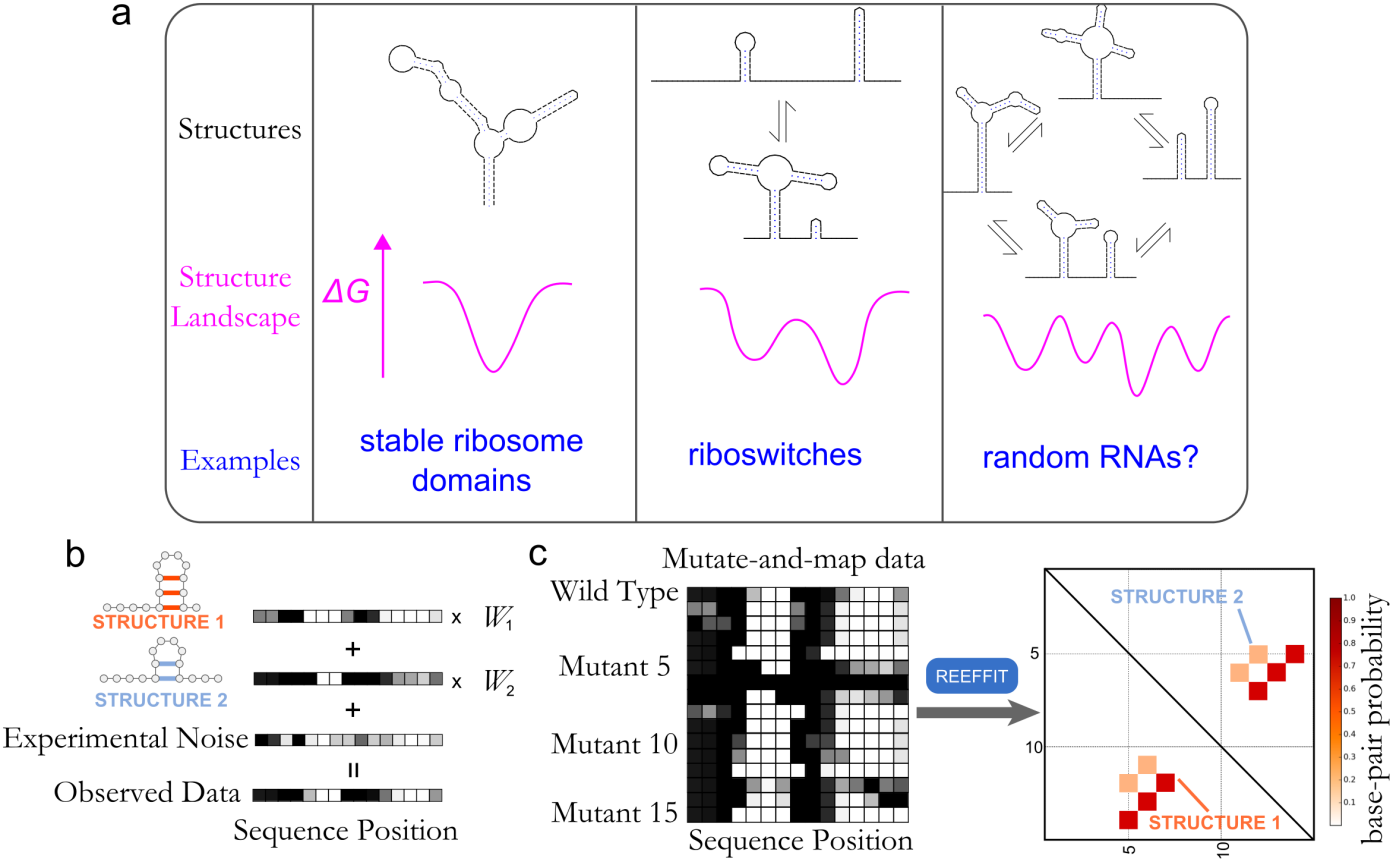
(a) RNA molecules can have diverse structural landscapes that are linked to their biological functions. Some structural landscapes, such as those for stable ribosome domains, can have a single, stable structure that is critical for function. Others may have been selected to have an equilibrium of two or three structures, such as riboswitches, that enables them to fine tune their response to small molecules or macromolecular partners. There may be other RNAs with a heterogeneous landscape, such as random RNA sequences, that may require this heterogeneity for function. (b) Chemical mapping (footprinting) experiments for probing RNA structure can be conceptualized as linear combinations of the underlying structures in the RNA’s structural ensemble. The chemical mapping profiles of an ensemble of two structures, represented here as one-dimensional heat maps, are scaled by their respective Boltzmann weights, *W_1_* and *W_2_*, and added together with experimental noise to form the observed chemical mapping pro?le of an RNA. (c) Mutate-and-map (M^2^) experiments are measurements of perturbed versions of an underlying structural landscape. A novel method, REEFFIT uses a blind source separation framework to automatically reconstruct this landscape by joint estimation of hidden reactivity profiles and fractions for a set of structures.

RNAs are deeply involved in gene expression, gene regulation, and structural scaffolding and are forming the basis of novel approaches to control these processes [1–3]. Several of RNA’s natural and engineered roles rely on its ability to fold into and interconvert between multiple functional structures. Ribozymes, riboswitches, and protein-complexed RNAs transition between several states to detect and respond to small molecules and other macromolecules; to proceed through numerous steps of RNA splicing reactions; to initiate, catalyze, and proof-read protein translation; to activate logical circuits in cells; and to package, release, and replicate RNA viruses [4–9]. The number of structures and equilibrium fractions that constitute these ‘dynamic structure landscapes’ are linked to the biological function of the RNA (Fig 1a). Rationally dissecting and re-engineering these landscapes depends on knowledge of the alternative states of an RNA’s structural ensemble [10,11].

Empirical portraits of such landscapes are missing for the vast majority of natural and engineered RNAs and it is unclear whether a rich multi-state landscape is a property specially selected by evolution or an intrinsic feature of RNA that can arise without explicit design or selection. Watson-Crick RNA secondary structure landscapes have been computationally predicted using dynamic programming techniques for decades [12,13] and many RNAs are predicted to form multiple structures at equilibrium, with ‘non-native’ helices reaching populations of 25% or greater [12–14]. In some cases, the alternative structures—and not the dominant structure—harbor motifs that are recognized by protein or small molecule partners [15–17]. However, difficulties in treating non-canonical interactions render these predictions inaccurate. Indeed, some studies have suggested that conformational switches are special hallmarks of biological function rather than an intrinsic feature of generic RNA sequences [18–21].

Unfortunately, the few experimental techniques that can validate or refute multi-state models are costly and difficult. For example, single molecule methods have been successful at revealing rarely populated RNA states but have not provided enough information to infer their structures [22]. Powerful insights have come from advances in nuclear magnetic resonance (NMR) spectroscopy [23] but require focused technical expertise, expensive infrastructure, and RNA targets with limited structural heterogeneity. In contrast, RNA chemical mapping, or footprinting, is a simple class of techniques that can achieve single-nucleotide resolution structural data for any RNA [24,25]. For RNAs with multiple states, however, these chemical mapping data give ensemble averages over all states, leading to a dramatic loss of information compared to what would be needed to resolve the RNA’s dynamic structure landscape and to make testable predictions (Fig 1b, see also refs. [20,26]).

While developing experiments that couple systematic mutagenesis with chemical mapping (mutate-and-map, or M^2^), we observed that single mutations can produce dramatic changes in chemical mapping data throughout an RNA sequence, with several mutations often giving the same alternative pattern [27,28]. We hypothesized that these perturbed patterns correspond to the reweighting of the structural ensemble of the RNA so that alternative component states dominate the chemical mapping data (Fig 1c). We have recently shown that such alternative structures can be inferred after expert inspection of M^2^ measurements and extensive compensatory rescue studies in an *E*. *coli* 16S ribosomal RNA domain [29]. Reasoning that such landscape dissection might be fully automated through the use of blind source separation algorithms, we have now developed an analysis framework called the RNA Ensemble Extraction From Footprinting Insights Technique (REEFFIT; see Fig 1c).

Here, we sought to use M^2^-REEFFIT to determine whether complex landscapes could arise in artificial RNAs without explicit natural selection or design. Prior techniques for detecting alternative structures, including covariation-based methods [19] and the recent RING-MaP method [20], have not been benchmarked in cases with well-characterized landscapes and may be biased towards or against alternative structures. Therefore, we developed three tests for M^2^-REEFFIT landscape dissection. First, a benchmark on simulated M^2^ data for 20 natural non-coding RNA sequences provided ‘gold standard’ reference results allowing unambiguous assessment of accuracy. Second, we applied M^2^-REEFFIT to four experimental test cases involving biological and artificial RNAs that had been previously characterized in detail by NMR or chemical mapping. For these cases, M^2^-REEFFIT recovered the landscapes defined through prior expert analysis. Third, we developed a validation approach based on stabilizing and experimentally testing predicted structures by multiple mutations. With these benchmarks and methodological developments, we used M^2^-REEFFIT to demonstrate that an imperfectly designed riboswitch and a randomly generated RNA sequence each form at least three structures, including states that could not be predicted from computational modeling alone.

## Results

### Dissecting landscapes *de novo* with M^2^-REEFFIT

To leverage the signals of stabilized alternative structures present in M^2^ measurements, we developed a new analysis framework, REEFFIT, to simultaneously infer multiple structures and their population fractions across the mutant RNAs (see Methods). We first applied M^2^-REEFFIT to infer structure landscapes for an *in silico* benchmark of 20 sequences drawn from the Rfam database of non-coding RNA molecules. We simulated M^2^ data by randomly choosing up to 200 suboptimal structures in the ensembles of the wild type sequence and of all variants that mutate a single nucleotide to its complement, as are typically probed in M^2^ experiments. We then simulated these structures’ respective SHAPE reactivity profiles using known reactivity distributions. To mimic inaccuracies in available energetic models, we re-weighted the ensemble randomly by introducing Gaussian noise (mean of 0 kcal/mol and standard deviation of 1 kcal/mol) to the predicted free energies. The resulting landscapes ranged from single dominant structures (e.g. cases RF00027 and RF00301) to more complex scenarios with two or three states (e.g. cases RF01300 and RF00051) to multi-state RNAs with more than three states present at non-negligible fractions (e.g. cases RF01274 and RF01125; Fig A in S1 Text gives five examples examined in detail in the Supporting Results, Fig B in S1 Text gives results for the entire benchmark, and the figures in S2 Text contains detailed Figs for all benchmark cases).

Over the entire 20-RNA benchmark, REEFFIT was able to consistently detect the presence of dominant and alternative helices (Supporting Results and Figs A-D in S1 Text). We set a criterion for helix detection that the fitted population should be larger than the population error estimated from bootstrapping, i.e. the signal-to-noise ratio should be greater than one. With this criterion, REEFFIT achieved 94.6% sensitivity over helices present with at least 3 base pairs and at least 25% population, corresponding to a false negative rate (FNR) of 5.4%. The false discovery rate (FDR) was low as well, at 9.7% (see Table 1 and Table A in S1 Text; full precision-recall curves given in Fig B in S1 Text). Without data, the error rates were substantially worse, by three-fold and two-fold, respectively (FNR of 18.0% and FDR of 23.3%). Values for base-pair-level error rates were similar to helix-level error rates (Table B in S1 Text). Error rates for wild type sequences alone (i.e., excluding single-nucleotide mutants) were higher but also showed a strong improvement in REEFFIT ensembles compared to ensembles modeled with no data (FNR of 15.2%, FDR of 15.2% for REEFFIT; FNR of 24.5% and FDR of 25.4% without data, see Table C in S1 Text). Use of only wild type data and no mutants, as would be carried out in conventional chemical mapping measurements, gave high error rates similar to landscapes modeled without data (FNR of 22.7% and FDR of 25%, see Table D in S1 Text and an example in Supporting Results and Fig D in S1 Text), confirming the necessity of M^2^ data for accurate landscape dissection. Further systematic checks and use of ViennaRNA [30] initial models and benchmark evaluation using helix-wise RMSD are given in the Supporting Results in S1 Text and Tables E, F, and G in S1 Text.

**Table 1:** Performance results for M^2^-REEFFIT landscape dissection on a 20 RNA benchmark from the RFAM database.

		No data^a^		M^2^-REEFFIT^a^	
Rfam ID	No. Helices^a^	TP	FP	TP	FP
RF00031 SECIS	3.02	2.48	1.47	2.88	0.09
RF00051 mir-17	5.35	4.98	0.18	5.31	0.42
RF00014 DsrA	4.44	3.88	0.56	4.32	0.93
RF01092 GP_knot2	3.40	2.60	1.47	3.26	0.56
RF00066 U7	3.31	1.77	2.31	2.71	1.26
RF01300 snoU49	3.56	2.88	1.46	3.49	0.76
RF01139 sR2	2.61	2.29	2.34	2.50	1.34
RF01274 sR45	2.34	1.96	1.02	2.13	0.82
RF01297 sR40	3.42	2.26	2.95	3.29	2.21
RF00555 L13_leader	2.96	2.91	0.51	2.93	0.28
RF00775 mir-432	4.98	4.35	1.35	4.78	1.78
RF00173 Hairpin	2.64	2.28	0.96	2.47	0.79
RF00108 SNORD116	2.03	1.32	1.95	1.86	1.51
RF00570 SNORD64	3.69	2.80	0.66	3.39	0.59
RF01301 snoR4a	2.67	2.20	2.35	2.56	1.28
RF01125 sR4	2.63	2.05	0.36	2.47	0.20
RF00436 UnaL2	1.79	1.79	0.21	1.79	0.18
RF01151 snoU82P	1.90	1.24	1.30	1.76	1.06
RF00027 let-7	3.45	3.27	1.69	3.30	1.88
RF00042 CopA	6.22	5.93	0.32	6.19	0.30
	FDR	23.3%		9.7%	
	FNR	18%		5.4%	

^a^Values are averaged across all variants probed (wild type RNA and single mutants).

### Experimental tests on RNAs previously characterized by M^2^ and expert analysis

**Fig 2:**
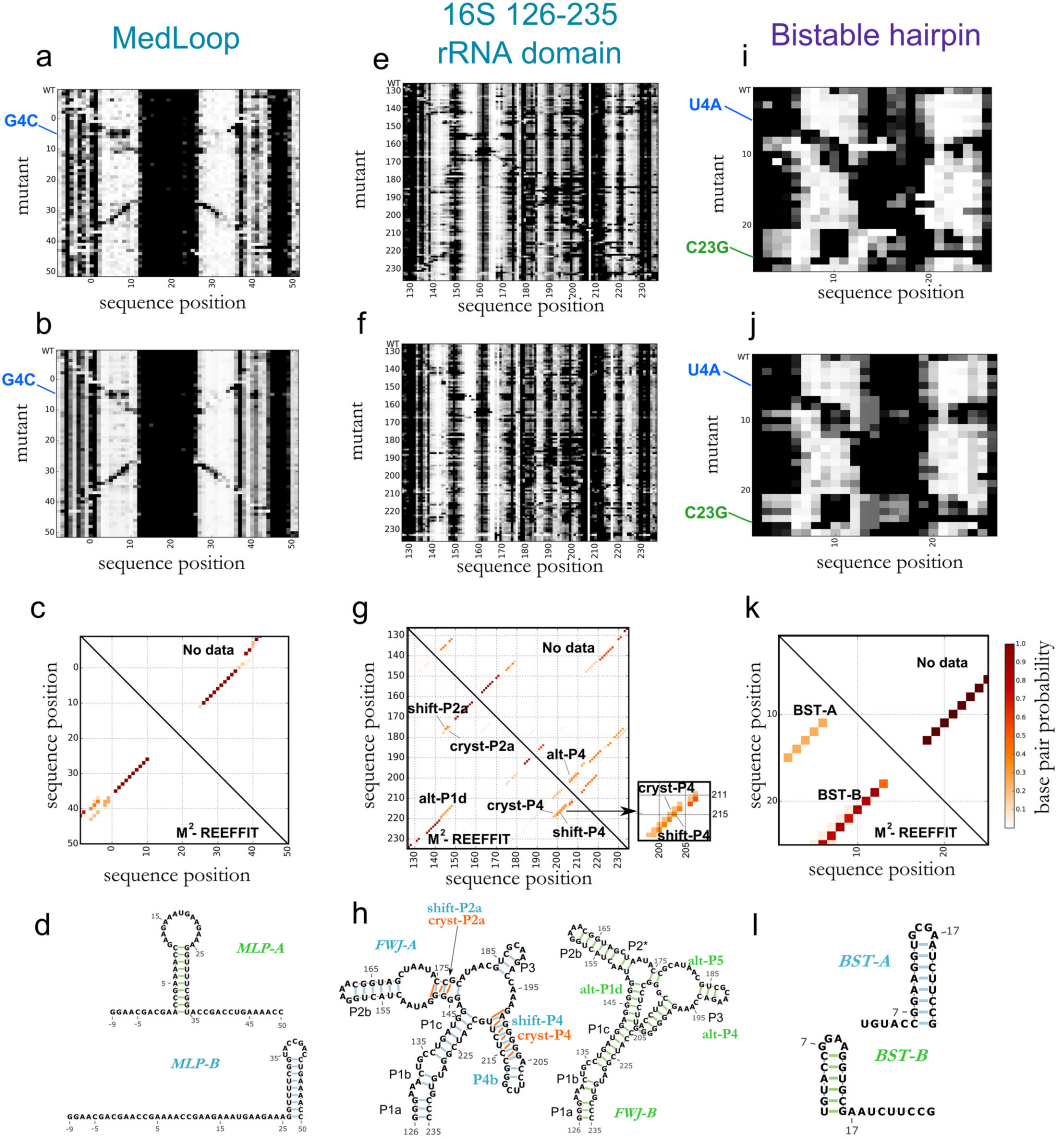
Landscape dissection of four diverse RNA systems from experimental mutate-and-map (M^2^) data. (a) M^2^ measurements for the MedLoop artificial sequence (given to REEFFIT as input); (b) REEFFIT bootstrapped fits; (c) base pair probability matrices of the structures in the wild type landscape (upper triangle: base pair probability values using no data, lower triangle: REEFFIT calculated base pair probabilities); and (d) structure medioids found *de novo* by REEFFIT. M^2^-REEFFIT analysis are also presented for (e-h) a four-way junction domain of the 16S rRNA, (i-l) an artificial bistable hairpin.

Success in the above *in silico* benchmark suggested that REEFFIT would accurately recover prior analyses of experimental M^2^ data sets, such as the MedLoop RNA (Fig 2a–2d). This RNA was previously designed to exhibit a single, stable 10 base pair helix with a 15 nucleotide loop (MLP-A, Fig 2d) [27]. A few mutations were observed to give a clearly distinct chemical mapping pattern and predicted computationally to fold into an alternative structure (MLP-B in Fig 2a; see e.g. G4C in M^2^ data) but not expected to be strongly populated in the wild type sequence. Automated REEFFIT modeling of the MedLoop M^2^ data recovered both the dominant structure MLP-A and the alternative state MLP-B, and bootstrapped uncertainties gave bounds on the frequency of MLP-B in the wild-type sequence (4±4%, see mutant-wise state fractions in Fig Fa-Fc in S1 Text). Thus, REEFFIT was able to explain rearrangements of mutants into an alternative state, but at the same time did not over-predict its presence in the wild-type sequence (Fig 2c).

As a more difficult test, we applied the algorithm to the 126–235 region of the *E*. *coli* 16S ribosomal RNA. In protein-bound ribosome crystals, this domain forms a four-way junction with helices P1a-c, P2a-b, P2*, P3, and P4a-b, but its solution state has been controversial [29] (Fig 2e–2h). Conventional SHAPE-guided analysis suggested loss of P2a and P4, and formation of alternative helices alt-P1d and alt-P4 [31] (Fig 2h, green structure). However, M^2^ and compensatory rescue experiments gave no evidence for the SHAPE-based model but instead recovered the dominant solution structure to be the holo-ribosome conformation, except for a register shift in P4a (called shift-P4a); the crystallographic P4 register was also detected as a 20±10% ‘excited state’ [29]. In agreement with this detailed analysis, automated REEFFIT analysis of the 126–235 RNA M^2^ data also returned helices P1a-c, P2*, P2b, and P3 with high population fractions (>80%, Fig 2g). Importantly, REEFFIT recovered an admixture of P4 (21±16%) and shift-P4 (60±23%) in a 3:1 ratio, in agreement with prior analysis (Fig 2g, magnification); RNAstructure calculations or use of wild type SHAPE data alone assigned negligible probability or highly uncertain population fractions to these helices, respectively (Fig 2g). The population of alternative helices alt-P1d and alt-P4 were found to have low populations and high errors (see Supporting Results in S1 Text). Further, a refined REEFFIT analysis including data for compensatory rescue double mutants recovered, with conservative error estimates, prior expert analysis (see Supporting Results and Fig E in S1 Text), illustrating the automation of modeling of even a complex RNA structure landscape.

### Experimental tests on RNAs with NMR-characterized landscapes

Encouraged by REEFFIT’s performance in previous test cases for chemical mapping, we sought tests involving fully independent experimental characterization by NMR. We first investigated a bistable RNA sequence whose landscape was dissected by Höbartner and colleagues by decomposing its NH…N ^1^H NMR spectrum into a weighted sum of two states forming different hairpins (here called BST-A and BST-B) [32]. As expected, M^2^ measurements gave clear evidence for two distinct chemical mapping profiles, reflecting the bistable nature of this RNA (Fig 2i–2l). One profile, consistent with BST-A appeared in variants with mutations in the 5´ end of the sequence, such as U4A. Another profile, consistent with BST-B, was revealed by mutations in the 3´ end such as C23G, which would destabilize the BST-A hairpin (Fig 2i). Both structures were recovered by REEFFIT analysis, with population weights of 73 ± 11% and 26 ± 9%, for BST-A and BST-B respectively, in agreement with the previously reported fractions measured by NMR (70 ± 5% and 30 ± 5%, respectively) and correcting the erroneous weights predicted with no data (99% and <1%, respectively).

**Fig 3:**
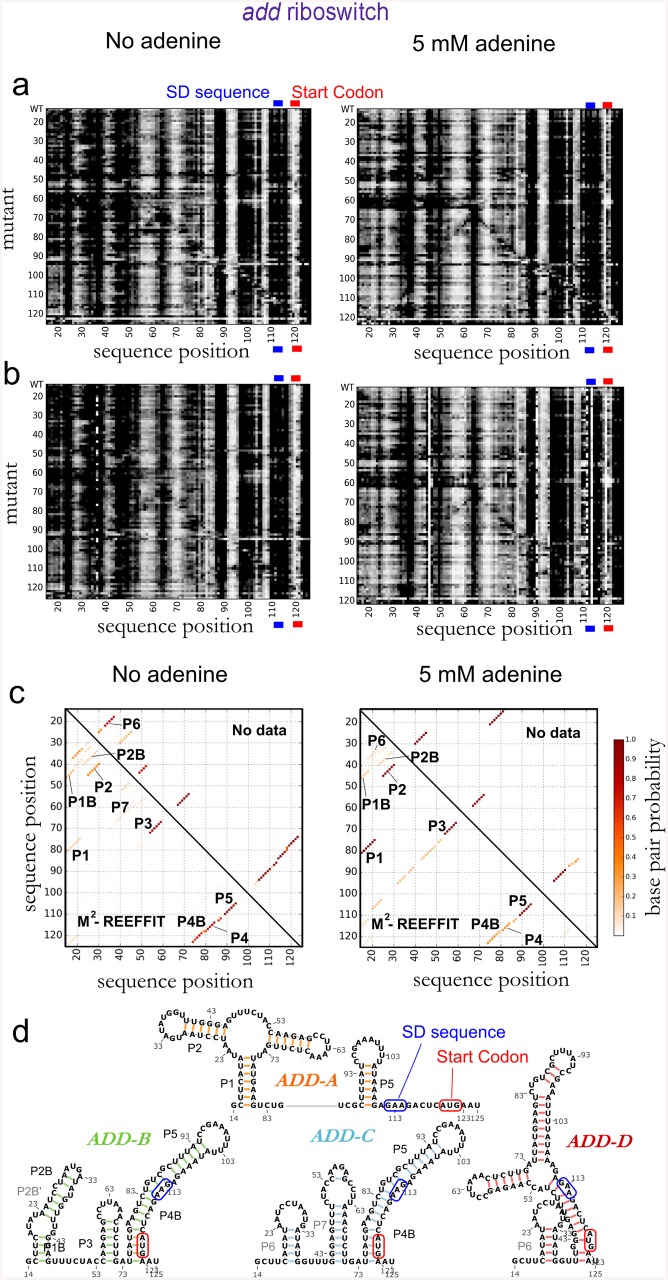
Landscape dissection of a segment of the *V. Vulnificus add* adenine riboswitch in 0 and 5 mM adenine. (a) M^2^ measurements in 0 and 5 mM adenine; (b) REEFFIT bootstrapped fits; (c) base pair probability matrices of the structures in the wild type landscape (upper triangles: base pair probability values using no data, lower triangles: REEFFIT calculated base pair probabilities); (d) structure mediods of the structural ensemble found by REEFFIT. In (a-b), The Shine-Dalgarno (SD) ribosome-binding sequence was observed to be SHAPE-reactive even in the absence of adenine; while consistent with this region's unpaired status without and with ligand in prior NMR studies, our studies indicate a more dramatic switch in this region for more complete *add* riboswitch constructs and will be reported elsewhere. See Supporting Tables H-I in S1 Text for RNA sequences and fit summaries.

We then challenged M^2^-REEFFIT with a more complex test case: a *Vibrio vulnificus* adenosine deaminase (*add*) mRNA riboswitch (Fig 3a–3d) that, in response to the ligand adenine, exposes start site segments (Shine-Dalgarno sequence and/or AUG start codon) to promote mRNA translation. In a detailed NMR study, spectra in ligand-free conditions fit well to a model with two states, apoA (~30%, with helices P1, P2, P3, P4, and P5) and apoB (~70%, with P1B, P2B, P3, an extended P4 called P4B, and P5). Addition of adenine ligand resulted in spectra dominated by a state holo with P1, P2, P3, and P5 and perturbed chemical shifts consistent with adenine binding to the aptamer [17]. While three states gave the simplest model for the prior data, more complex multi-state models were consistent as well and would be generally predicted from RNA secondary structure calculations [12,13]. We focused on whether M^2^-REEFFIT could recover the base pairs of P1, P2, P1B, P2B, P3, P4, P4B, and P5, which were unambiguously determined through NOE spectroscopy and model construct comparisons. Even in the absence of adenine, our M^2^ measurements of the RNA suggested the presence of at least two distinct structures that protect or expose the mRNA start site (AUG at nts 120–122) and, in an anticorrelated manner, expose or protect segments in the aptamer region (e.g., nts 53–60 and 66–72), respectively (M^2^ data in Fig 3a). In addition to observing these different states upon mutation, addition of 5 mM adenine induced clear changes in the M^2^ data, including protections in the aptamer and consistent exposure of the AUG start codon in most mutants (M^2^ data in Fig 3a, bottom;). While consistent with this region’s unpaired status without and with ligand in prior NMR studies, our studies indicate a more dramatic switch in this region for more complete add riboswitch constructs and will be reported elsewhere.

REEFFIT analysis gave excellent fits to these *add* riboswitch M^2^ data (Fig 3b), automatically detecting the presence of P1 (14±10%), P2 (37±15%), P3 (86±18%), P4 without extension (69±10%), the extension P4B at lower population (36±6%), and P5 (94±18%) in the absence of adenine, in agreement with the apoA and apoB model from NMR (Fig 3c–3d); and recovering a holo state with P1, P2, P3, P4, and P5 dominating in 5 mM adenine (see Supporting Methods in S1 Text for treatment of ligand-bound structures). In ligand-free conditions, the REEFFIT analysis also gave several alternative helices in the P1/P2 region, including P1B and P2B (20±7%), consistent with the NMR-detected features in the apoB structure. The M^2^ data were critical in making these detections; RNAstructure calculations and use of wild type SHAPE data alone assigned negligible (<5%) probability to P1, P1B, P2B, and the possibility of P4B shortening to P4 (Fig 3c–3d). Coarse clustering of REEFFIT structures returned four states in which the NMR-modeled apoA was recovered as the cluster medioid of one state, ADD-A, and apoB was recovered as a medioid in another, ADD-B, albeit with an additional helix, P4B (Fig 3c). Structures with alternative helices to P1, P2, P1B, and P2B in the 5´ region clustered into states ADD-C and ADD-D. The population fractions of these helices, as well as a set of P6 and P7 helices not detected in NMR experiments, were low; these features were appropriately flagged as uncertain from bootstrapping analysis (e.g., 21±15% for the most populated helix of this kind, P6). Overall, the M^2^-REEFFIT results successfully recovered NMR-detected helices for this adenine riboswitch sequence, including heterogeneous structure in the 5´ region, dynamics in the P4 region, and rearrangements on adenine addition.

### Assessing if artificial sequences exhibit complex RNA landscapes

**Fig 4:**
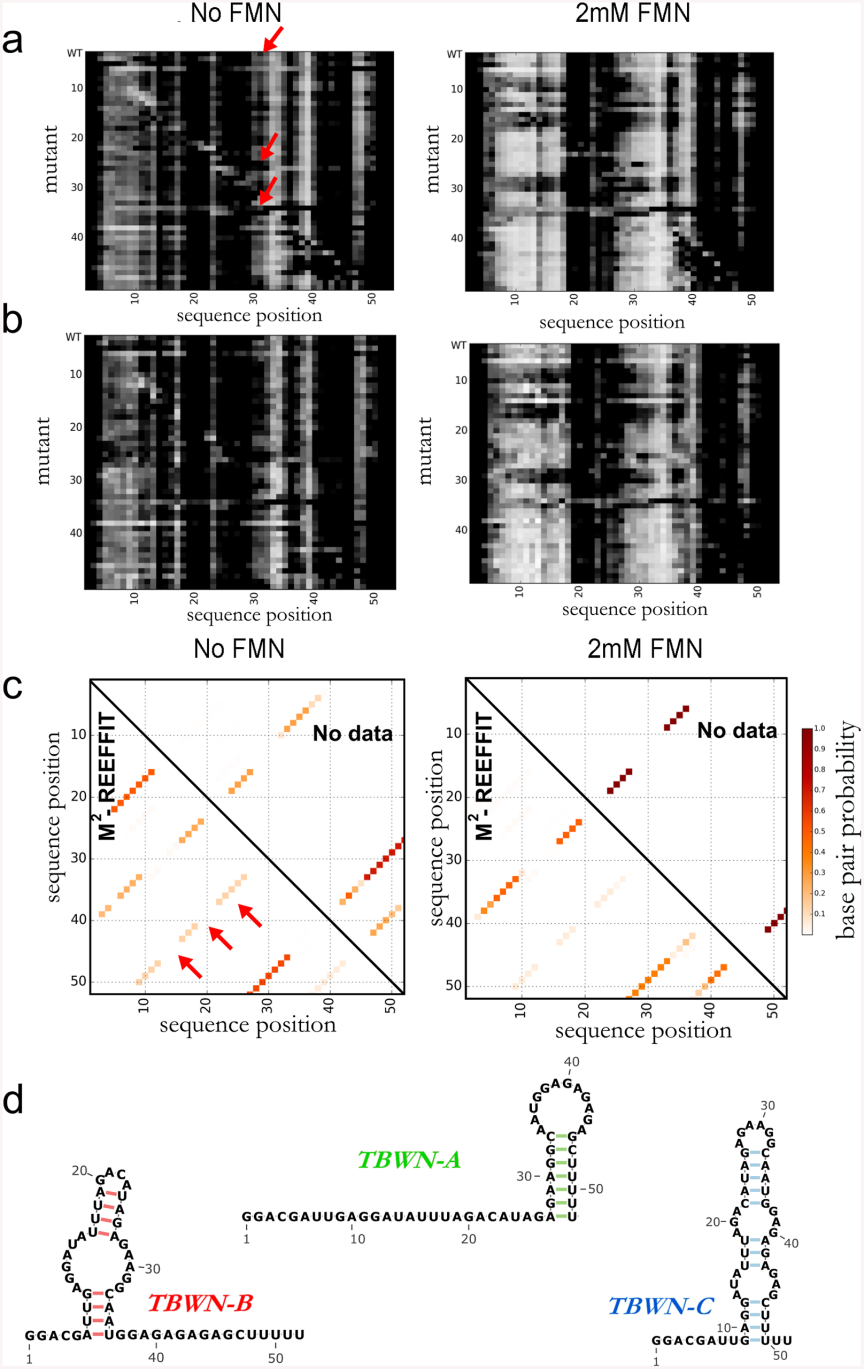
Landscape dissection of a designed FMN riboswitch with a structural discrepancy, the Tebowned RNA. (a) M^2^ data, (b) REEFFIT fit, and (c) inference of the Tebowned structural landscape, which is predicted by REEFFIT to fold into (d) three prevalent structures TBWN-A, TBWN-B, and TBWN-C. Presence of TBWN-C is marked with red arrows in (a) and (c).

**Fig 5:**
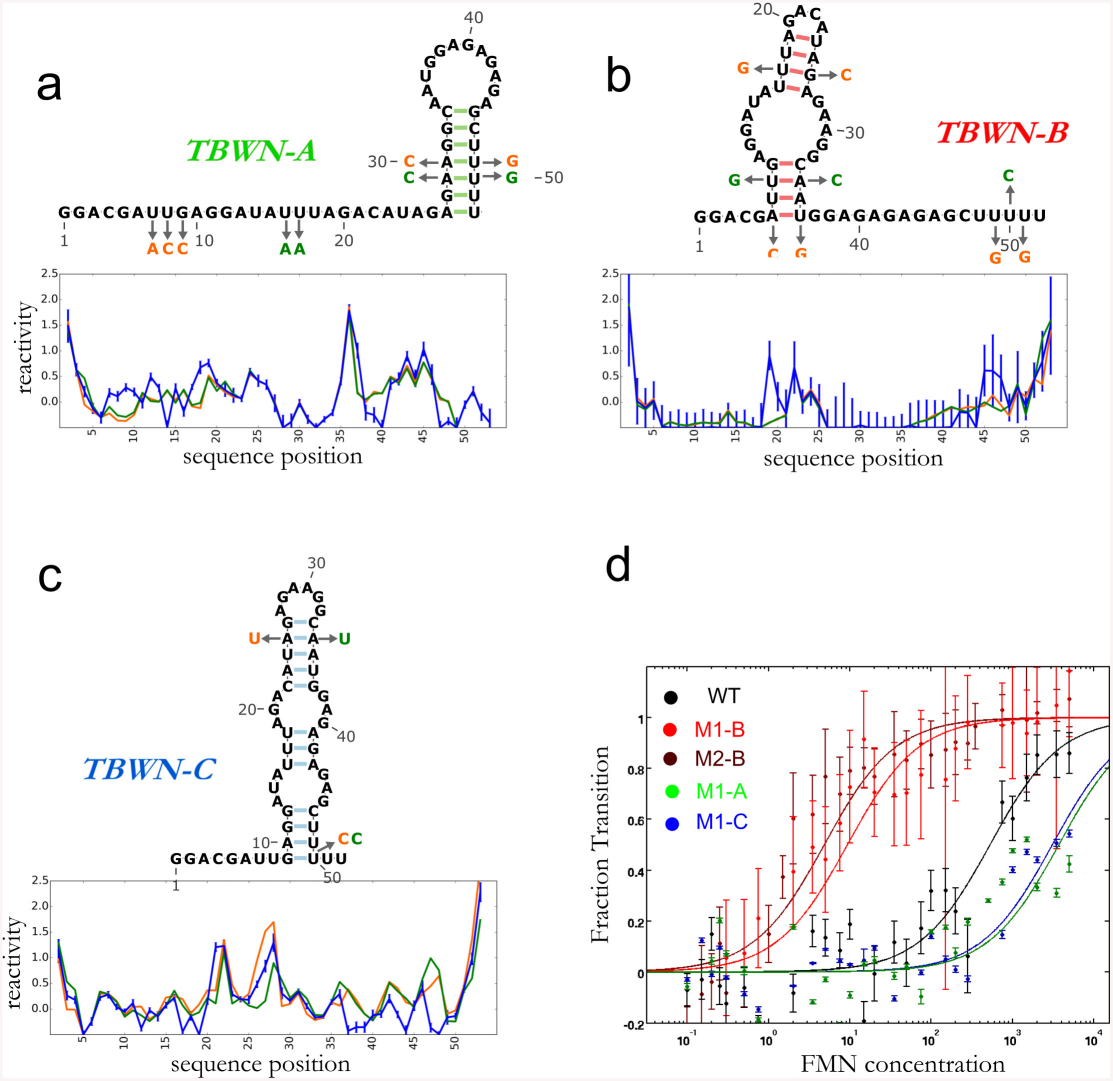
Comparison between reactivities predicted by REEFFIT and mutants designed to stabilize the (a) TBWN-A, (b) TBWN-B, (c) TBWN-C structures are given below each state (arrows in the structures mark mutations); each plot shows the measured reactivates for each pair of mutants (orange and green) and predicted REEFFIT reactivates for the corresponding state (blue with error bars). (d) Determining FMN dissociation constants for stabilizing mutants of the TBWN-A, TBWN-B, and TBWN-C structures of the Tebowned FMN switch using the LIFFT HiTRACE toolkit (showing data for residue 15, see Supporting Fig I in S1 Text) are shown below the mutants.

After validation of M^2^-REEFFIT on diverse computational and experimental test cases, we used the method to estimate whether complex landscapes might arise in artificial RNA sequences without explicit design or selection. First, we analyzed the folding landscape of an imperfect RNA switch, ‘Tebowned’, that was designed to convert between two states upon flavin mononucleotide (FMN) binding (Figs 4 and 5) in early rounds of a riboswitch design puzzle in the Eterna massive open laboratory [33]. While the chemical mapping pattern of this RNA changed upon binding FMN, the measurements for the unbound state did not match the desired unbound structure, particularly near nucleotide A30 (red arrows in Fig 4a); this region should have been paired but instead was measured to be reactive. *A priori*, we could not distinguish whether this discrepancy was due to an incorrect balance of the two target bound and unbound structures or if there were other unexpected structures involved. To elucidate the discrepancy, we acquired M^2^ data for the Tebowned RNA. We first used REEFFIT to fit the M^2^ data using only the two desired structures, but this fit did not capture several features, such as the exposure at A30 (Fig G in S1 Text). In contrast, a global ensemble fit adequately captured these features (Fig 4a–4b, and Fig Gb in S1 Text). REEFFIT automatically clustered the modeled ensemble into three states: TBWN-A (56 ±16%) and TBWN-B (27 ± 12%), matching the desired switch structures, and a third structure TBWN-C present at 17 ± 11% fraction (red arrows in Fig 4a and Fig Ha-Hc in S1 Text). The unexpected state TBWN-C exhibits an apical loop around nucleotide A30, explaining the observed discrepancies in this region for the wild type RNA, and harbors a purine-rich symmetric loop that may be significantly stabilized compared to the energy assumed in current nearest-neighbor models [34]. Other helices were discovered to be populated at non-negligible fractions in the analysis, but were deemed uncertain (signal-to-noise ratios less than 1) from bootstrapping analysis.

The REEFFIT-inferred populations of TBWN-B and TBWN-C were low (<30%). Therefore, to further test the presence of at least three states, we sought to compare their modeled REEFFIT reactivity profiles to actually measured reactivities for these states. To achieve this comparison, we designed mutants that specifically stabilized helices in each of the TBWN-A, TBWN-B, and TBWN-C structures (Fig 5a–5c). For each state, the reactivities of these state-isolating mutants agreed with each other within experimental error and were approximated well by REEFFIT’s predicted profile, providing independent confirmation of the modeled ensemble, including the unanticipated third state TBWN-C. Additional evidence for the accuracy of REEFFIT’s predictions was revealed by each of the stabilizing mutants’ FMN binding affinities: the TBWN-B and TBWN-A/TBWN-C mutants enhanced and worsened ligand binding, respectively, as expected (Fig 5d, and Supporting Results and Fig I in S1 Text). We emphasize that the TBWN-C state would have been difficult to propose and then validate without automated REEFFIT analysis, given its negligible predicted population in secondary structure prediction calculations without M^2^ data (Fig 4c).

**Fig 6:**
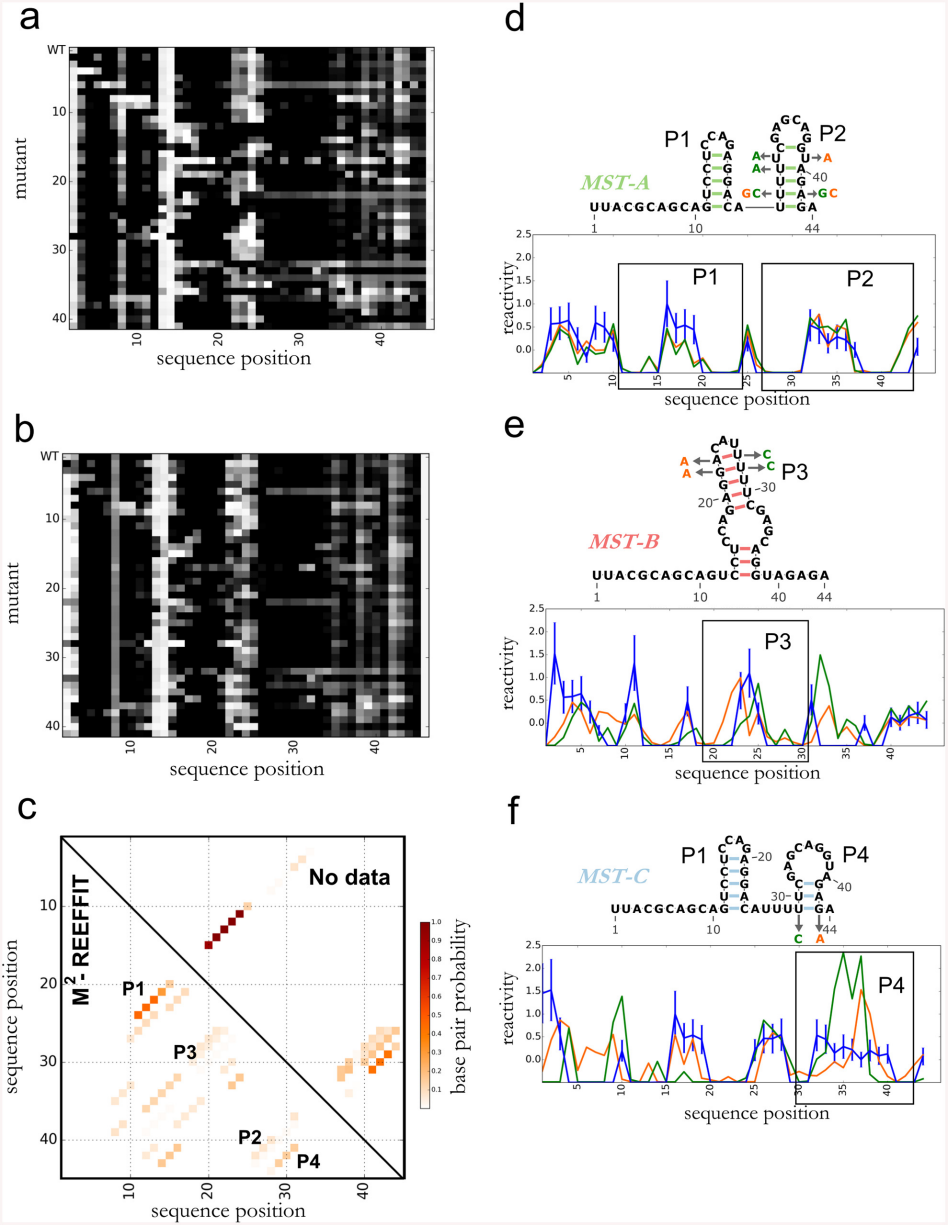
Landscape dissection of a random RNA sequence, the M-stable RNA. (a) M*2* data, (b) REEFFIT fit, and (c) inference of the M-stable structural landscape, showing one helix, P1, present at almost 50% as well as numerous other helices (P2, P3, and P4) present at non-negligible fractions. (d-f) State medioids for (d) MST-A, (e) MST-B, and (f) MST-C, and comparison between reactivities predicted by REEFFIT and mutants designed to stabilize each state medioids.

As a second test case with a previously unknown structural ensemble, we tested whether randomly generated or scrambled RNA sequences tend to fold into multiple disparate structures at equilibrium—a long-standing hypothesis fundamental to understanding RNA evolution, put forth by several *in silico* studies [35,36] and an experimental study that could not deconvolve the structures [37]. We carried out M^2^-REEFFIT for a randomly generated sequence, called here the M-stable RNA (Fig 6). Based on simulations, the structural ensemble of the construct was expected to consist mainly of a simple hairpin (P1 in MST-A in Fig 6d, see top triangle of the Fig 6c) but with at least two other structures becoming more stable than MST-A upon single mutations. The experimental M^2^ measurements were complex, with different mutants giving disparate protection patterns even in segments that appeared highly reactive (and seemingly unstructured) in the wild type RNA. As a first check on the number of states, REEFFIT fits assuming only 2 or 3 states missed many features observed in the data, including extended segments of changed chemical reactivity in several mutants (Fig J in S1 Text). However, the REEFFIT global ensemble fit successfully modeled the M-stable data and suggested an ensemble with many more weakly populated helices than RNAstructure’s estimate (compare bottom and top halves of Fig 6c). For visualization, we clustered these heterogeneous component structures into three states, MST-A, MST-B, and MST-C (see Fig 6d–6f and Fig Hd-Hf in S1 Text). Analogous to the case of the Tebowned switch, we tested the REEFFIT prediction of these alternative states by designing mutations to stabilize the medioid structures of each cluster (Fig 6d–6f). These mutants gave reactivities in agreement with predictions for MST-A and MST-C, supporting the inference of those structures. For MST-B, the state-stabilizing mutants gave reactivity profiles that did not exactly match each other, suggesting residual heterogeneity of structure; the profiles were nevertheless closer to the REEFFIT-predicted MST-B profile than wild type reactivities. These results corroborate the M^2^-REEFFIT model that the M-stable random RNA has a complex landscape with at least three structures, and likely significantly more heterogeneity, detectable upon unbiased nucleotide mutation.

## Discussion

The structure landscapes of natural and newly designed RNA molecules underlie their biological behaviors, but these landscapes’ complexities are largely uncharacterized [11]. Current experimental techniques used to probe these ensembles at nucleotide resolution require significant infrastructure investment and expert intuition. We have presented M^2^-REEFFIT, an unbiased strategy based on readily acquired chemical mapping measurements that detects dominant and alternative states of RNA structure landscapes in the ensemble perturbations induced by single-nucleotide mutations, with conservative estimates based on bootstrapping.

Due to the challenges inherent in estimating a full ensemble of secondary structures rather than a single best-fit model, we have invested significant effort into benchmarking M^2^-REEFFIT and its uncertainty estimates. We confirmed the accuracy of REEFFIT and its uncertainty estimates in M^2^ simulation data with known ‘ground truth’ landscapes and in experimental data of RNAs whose landscapes were previously studied by chemical mapping or NMR experiments. We then applied REEFFIT to investigate if artificial sequences could present complex landscapes of alternative structures without specific design in two model systems whose ensemble behaviors were refractory to prior tools. We first traced a structural discrepancy in an artificial flavin-mononucleotide-binding switch to a significant population of an unexpected state. We then tested *in silico* predictions for the complex structural landscape of random sequences by obtaining the first experimentally derived landscape model of a randomly generated sequence, the M-stable RNA. For all cases, we tested REEFFIT’s predictions through independent experiments, including comparison to independent experimental methods and effects of stabilizing mutations and ligand binding.

Beyond the inference of structural landscapes of single sequences, the high-throughput nature of M^2^-REEFFIT makes inference of the landscapes of multiple sequences related through their presumed or engineered function possible. For example, when dissecting structural features of sequences selected for binding efficiencies to molecular partners through *in vitro* selection or when analyzing the role of conserved structural motifs in diverse RNA sequences, a full portrait of their landscapes may reveal the interplay of several states that is critical for function. As an example, one of our experimental test cases, the Tebowned FMN switch, illustrates that information relevant for function can be extracted from knowing the full landscape of the RNA. In this case, we have a ‘functional’ readout of a structural motif (the FMN binding motif) that is present at different fractions in different mutants of the M^2^ experiment. A full view of the ensemble yields an extra state, TBWN-C, whose interplay with the state that contains the binding motif, TBWN-B, is necessary for understanding FMN binding of the riboswitch and its variants.

Currently, uncertainties in the reactivities and energies of RNA motifs lead to RMSD errors in M^2^-REEFFIT state population fractions on the order of 10–15%, rendering the detection of states with lower population fractions difficult; these uncertainties may become poorer for longer RNA domains. Nevertheless, we expect that rapidly growing databases of rigorously standardized reactivity data [38,39] and of energetic parameters [40] for diverse RNA motifs will reduce these uncertainties. Furthermore, M^2^ experiments performed with other chemical probes, such as dimethyl sulfate, should provide powerful cross-validation data sets for testing inferred landscapes. Through the presented and related chemical mapping technologies, we therefore expect to have more routine visualization of the rich structural landscapes that appear to be pervasive in both functional RNAs and the generic sequences from which they evolve.

## Methods

### M^2^ measurements

The MedLoop RNA mutate-and-map data were obtained as part of the experimental pipeline of the Eterna massively parallel open laboratory [27],[41]. The MedLoop RNA and its mutants were generated through *in vitro* transcription of a pool of DNA constructs purchased from CustomArray. The RNA was probed with 1-methyl-7-nitroisatoic anhydride (1M7) in a folding buffer (see Table I in S1 Text for detailed folding conditions) using the MAP-seq protocol and sequenced. in a MiSeq sequencer. The resulting reads and were analyzed with the MAP-seeker software [41]. The Bistable hairpin, Tebowned switch, and M-stable RNAs as well as their respective complementary single-nucleotide mutants were constructed using PCR assembly, *in vitro* transcription, and probed with 1M7 as described previously [42]. Briefly, an assembly consisting of at most 60-nucleotide primers was designed to synthesize an *in vitro* transcription sample by PCR. DNA was purified with AMPure XP beads (Agencourt, Beckman Coulter) and *in vitro* transcribed for 3 hours. The resulting RNA was purified with AMPure XP beads, heated for 3 minutes at 90°C, cooled at room temperature for 15 minutes, and folded in folding buffer at room temperature for 1 hour (see Table I in S1 Text for detailed folding conditions for each RNA). Because we sought to probe the ensemble of the RNA with minimal interference from the 3´ unpaired sequence that we use as the primer binding site, we folded the RNA in the presence of the fluorescent primer attached to the oligo(dT) beads (Ambion) that we regularly use for purification. Folding in this condition sequesters any additional single stranded regions that may interfere with our sequence of interest. The RNA was then subjected to 1M7 mapping (5 mM final concentration), purified with the oligo(dT) beads, and reverse transcribed for 30 minutes at 42°C. Unmodified RNA controls were also included in the experiment. RNA was then degraded using alkaline hydrolysis and cDNA was purified, eluted in Hi-Di Formamide spiked with a fragment analysis ladder (ROX 350 standard, Applied Biosystems), and electrophoresed in an ABI 3150 capillary electrophoresis sequencer. The add adenine riboswitch M^2^ data was obtained similarly but folded under different conditions, in the NMR buffer of the previous study (50 mM KCl and 25 mM K_3_PO_4_, 10 mM MgCl_2_, pH 6.5) with or without 5 mM adenine for bound and unbound conditions, [17] For NMR folding conditions, we adjusted the 1M7 incubation time to 15 minutes instead of 3 minutes to account for the low pH. For the Tebowned FMN titrations, dimethyl sulfate (DMS) was used in lieu of 1M7 since it yields a readily seen signal change across FMN concentrations [33].

Electrophoretic traces were aligned, baseline subtracted, and normalized with the HiTRACE MATLAB toolkit [43]. 1M7 modification traces were quantified, background subtracted, and corrected for attenuation using 10X dilutions, the unmodified controls, and the pentaloop hairpins added at the ends of the constructs as reference [44]. For the Tebowned RNA, no pentaloop hairpins were added and we relied instead on the HiTRACE background subtraction routine overmod _and_background_correct_logL using unmodified controls. The lifft function from the LIFFT package [45] in HiTRACE was used to calculate the FMN binding dissociation constants for REEFFIT comparisons. Mutants with low signal were flagged as low quality and were not taken into account for the analysis.

### The RNA Ensemble Extraction From Footprinting Insights Technique (REEFFIT)

For a given mutate-and-map data set, REEFFIT infers the expected reactivity profile of each structure, the combination of structure population fractions (also denoted here as structure weights), and sequence-position-wise noise levels that best fit the data (see Figs 1b and 1c) using prior information on known chemical reactivity distributions, a weak prior based on an approximate secondary structure energetic model, and a well-defined likelihood function. To achieve this fit, we expanded Gaussian factor analysis, a standard blind-source separation technique, to include position-specific, non-negative, non-Gaussian priors for the expected reactivities and factor weights that depend on both structure and sequence position. Local perturbations due to mutations, including the release of base pairing partners induced by the single nucleotide changes, were also included in the statistical model (see section below “Handling local perturbations”). The model including these variables and parameters was fitted by REEFFIT using a maximum *a posteriori* (MAP) approximation. (A Bayesian simulation inference method sampling over the posterior distribution gave indistinguishable results at significantly greater computational expense and is not presented in detail here.). In this section, we present the basic idea behind the model. Then, in the following sections we introduce the priors used for each variable and parameter in the model. Finally, we incorporate these priors and present inferential steps used to fit the model to the data.

In a broad sense, for *m* chemical mapping measurements of *n*-nucleotide sequences, REEFFIT models the data, denoted here as *D* ^*obs*^ ∈ ℜ^*m*×*n*^, with a set of *r* secondary structures. The data are modeled as linear combinations of the structures’ reactivity profiles, denoted here as a matrix *D* ∈ ℜ^*r*×*n*^, with a weight matrix *W* ∈ ℜ^*m*×*r*^ plus Gaussian noise (see Fig 1b). Then, for each measurement *j* we have:

$$\begin{array}{c} D_{ji}^{obs}=\;\underset{s\;\in \;\text{structures}}{\sum }W_{js}D_{si}+\epsilon_{i}\\ \epsilon_{i}∼N\left(0,\Psi_{i}\right) \end{array}$$ (1)

The rows of the weight matrix *W* correspond to the population fractions of the structures in each measurement. Therefore, the corresponding weights for each measurement define a probability distribution and are non-negative and add up to one:

$$\begin{array}{c} \underset{s\;\in \;\text{structures}}{\sum }W_{js}\;=1,\;\forall j=1,\ldots ,m\\ \forall j,s\;W_{js}\geq 0 \end{array}$$ (2)

### Setting a prior on the “hidden” structure reactivities

In REEFFIT, *D* is a set of hidden variables since the isolated reactivity profiles of each structure are not typically available. We can impose a prior on each of these hidden profiles depending on their corresponding modeled secondary structure. Because a nucleotide’s chemical reactivity is reduced upon base pairing, a reasonable prior would force *D*_*si*_ to be small if *i* is paired in structure *s* and higher if it is unpaired. The needed priors are derived empirically from distributions of the reactivities of paired and unpaired nucleotides in the RNA Mapping Database (RMDB) of RNAs with known crystallographic structure [39,46]. Let *RMDB*_*U*_ and *RMDB*_*P*_ be the empirical RMDB unpaired and paired reactivity distributions respectively and *d* be some reactivity, we can then define a prior likelihood for *D*_*si*_ as:

$$RMDB_{si}\left(d\right)\;=\;\{\begin{array}{l} RMDB_{U}\left(d\right)\text{ if }i\text{ is unpaired in}\;s\\ RMDB_{P}\left(d\right)\text{ if }i\text{ is paired in}\;s \end{array}$$ (3)

Here, we equate reactivities to values given by SHAPE modifiers; to handle other modifiers, e.g. dimethyl sulfate, *RMDB*_*U*_ and *RMDB*_*P*_ can be replaced with the respective estimated distributions using unpaired and paired data of that modifier.

To simplify statistical inference, we approximated the RMDB reactivity distributions for paired and unpaired residues with exponential distributions, which has been found to be suitable for distributions of reactivities in unpaired regions [47]. We denote these approximations as:

$$\begin{array}{l} RMDB{*}_{P}(d)=\lambda_{P}\text{exp}\left(-\lambda_{P}d\right)\\ RMDB{*}_{U}(d)=\lambda_{U}\text{exp}\left(-\lambda_{U}d\right) \end{array}$$ (4)

Fitting these models to reactivity distributions for paired and unpaired residues in the RMDB gave scaling parameters of *λ*_*P*_ = 0.5 and *λ*_*U*_ = 0.2. We denote RMDB*_si_ as the resulting approximation for *RMDB*_*si*_ for structure *s* in position *i*, and *λ*_*si*_ as the corresponding scaling parameter, that is:

$$\lambda_{si}=\;\{\begin{array}{l} \lambda_{P}\text{ if }i\text{ is paired in s}\\ \lambda_{U}\text{ if }i\text{ is unpaired in s} \end{array}$$ (5)

### Handling local perturbations

Systematic experimental perturbations used to alter the RNA's structural ensemble may induce local changes that cannot be captured by the linear combination of the weights *W* and the hidden reactivities *D*. This is the case in M^2^ experiments, where mutations induce local perturbations in the underlying reactivities of each structure. To model these perturbations, we add a set of random variables that take the values of the change in reactivity of *D* at perturbed positions in order to account for the data. That is, we add a Δ*C*_*sji*_ variable for all sequence positions *i* that are in a set of perturbed sites for structure *s* in mutant *j*, *perturbed*(*s*,*j*). These perturbed sites are defined as positions lying at most one nucleotide away from the site of a mutation in mutant *j* or from a base pair that would be disrupted due to a mutation in structure *s* in mutant *j*. To simplify notation, we define *C* as containing the values of these perturbation variables at the relevant positions and mutants:

$$C_{sji}=\{\begin{array}{c} \Delta C_{sji}\text{ if }i\;\in \;perturbed(s,j)\\ 0\text{ otherwise} \end{array}$$ (6)

We set the prior distributions of Δ*C*_*sji*_ to a Gaussian approximation of differences in reactivities in M^2^ experiments available in the RMDB:

$$\Delta C_{sji}\sim N\left(0,1/\lambda_{perturbed}\right)$$ (7)

Fitting a normal distribution to the RMDB reactivity differences gave *λ*_*perturbed*_ = 2.

### Setting a prior for a regularized fit of the structure weights

Since REEFFIT potentially fits hundreds or even thousands of structures (see section below, “Building the structural ensemble and reducing model complexity”), we set a sparsity prior on the weight matrix *W*. Standard Laplacian (*l*_*1*_) sparsity cannot be imposed because the per-measurement weights are probability distributions and are constrained to sum to unity for each variant. We therefore imposed a “smooth sparsity” regularizer *λ*_*R*_ using a Gaussian distribution (*l*_*2*_), where weights found to be unimportant for the model are reduced to low, but typically non-zero, values.

$$W_{js}\sim N\left(0,1/\lambda_{R}\right)$$ (8)

To further avoid over-fitting weights, we encode a penalty that disallows dramatic deviations of computationally-predicted ΔΔ*G* values for each structure *s* between the wild type sequence and each mutant to values calculated from the RNAstructure package. For mutant *j*, let Δ*G*_*js*_ be the energy for structure *s* calculated by the efn2 program in the RNAstructure package [48], *k*_*B*_ the Boltzmann constant, and *T* the temperature at which the experiments were performed. We denote the RNAstructure weights at *(j*,*s)* as:

$$W_{0,js}=\frac{\text{exp}\left(-\frac{\Delta G_{js}}{k_{B}T}\right)}{\sum_{s^{\prime }}\text{exp}\left(\frac{\Delta G_{js^{\prime }}}{k_{B}T}\right)}$$ (9)

We then impose a Gaussian prior on wild type weights that drives their wild-type/mutant weight differences to be close to the RNAstructure values through a parameter *λ*_Δ_. Let wild type weights for each structure *s* are denoted as *W* ^WT^_*s*_ then:

$$\left|W^{\text{WT}}{}_{s}-W_{js}\right|\sim N\left(\left|W^{\text{WT}}{}_{0,s}-W_{0,js}\right|,1/\lambda_{\Delta }\right)$$ (10)

Values for *λ*_Δ_ and *λ*_*R*_ were obtained using a cross-validation strategy (see section below, *“*Building the structural ensemble and reducing model complexity”). These priors do not impose the probability distribution constraints of eq (2) on the measurement weights; instead, the probability distribution constraints are enforced during the optimization of the posterior function (see below).

### The REEFFIT statistical model

After defining the variables for modeling the data and their respective priors, we can write the complete REEFFIT model. Let *j = 1*,*…*,*m*, index over measurements. Then we write the model as:

$$\begin{array}{c} D_{ji}^{obs}=\;\underset{s\;\in \;\text{structures}}{\sum }W_{js}\left(D_{si}+C_{sji}\right)+\epsilon_{i}\\ \epsilon_{i}∼N\left(0,\Psi_{i}\right)\\ \begin{array}{cc} D_{si}∼RMDB{*}_{si}; & \Delta C_{sji}\sim N\left(0,1/\lambda_{perturbed}\right) \end{array}\\ \begin{array}{cc} W_{js}\sim N\left(0,1/\lambda_{R}\right); & \left|W^{\text{WT}}{}_{s}-W_{js}\right|\sim N\left(\left|W^{\text{WT}}{}_{0,s}-W_{0,js}\right|,1/\lambda_{\Delta }\right) \end{array}\\ \underset{s\;\in \;\text{structures}}{\sum }W_{js}\;=1\\ \forall j,\text{s }W_{js}\geq 0 \end{array}$$ (11)

Here, the hidden variables *D* and their perturbations Δ*C*_*sji*_ are encoded in *C*, while the parameters to estimate are *W* and the variances Ψ_*i*_. It is important to note that here, as in similar factor analysis models, all noise covariance matrices are assumed to be diagonal; that is, the measurements are independent of each other [49,50]. This assumption holds in the case of multiple chemical mapping measurements, since each measurement is carried out in different capillaries in capillary electrophoresis [51] or is based on Poisson distributed counts derived from separate single molecules in deep sequencing [41,52].

We used a hard expectation maximization (EM) algorithm to obtain MAP estimates for the values of the hidden variables and the model parameters. In hard EM optimization, the values for the hidden variables obtained in the E-step are maximum *a posteriori* estimates rather than expectations used in soft EM [50]. In standard factor analysis a soft EM is typically used: the E-step can be obtained in closed form by calculating the sufficient statistics for the likelihood function, which happen to be the first two moments of the posterior distribution of *D*: E[*D*|*D* ^obs^] and E[*DD* ^*T*^|*D* ^obs^] [49,50,53]. However, the non-Gaussian form of our priors for each *D*_*si*_, precludes a closed form for these statistics and we instead used a hard EM strategy. This strategy yielded results that were comparable with a much more computationally expensive soft EM procedure that used Markov Chain Monte Carlo (MCMC) to approximate E[*D*|*D* ^obs^] and E[*DD* ^*T*^|*D* ^obs^] in the E-step. For the M-step, we incorporated probability distribution constraints on *W* by casting the posterior maximization as a quadratic problem and solving it numerically.

### Maximum *a posteriori* estimation of the REEFFIT model

Given the REEFFIT factor analysis model (11) we want to calculate MAP estimates for *W* and each Ψ_*i*_ given the hidden variables *D*. The posterior function can be written as:

p(W,Ψ,D,C|Dobs)∝Likelihood×Prior of D×Prior of C×Prior of W=∏ i∈positionsj∈measurements1(2πΨi) 12exp(−12Ψi(Djiobs−∑s∈structuresWsj(Dsi+Csji))2)∏s∈structuresλsiexp(−λsiDsi)∏i∈perturbed(s,j)1(2πλperturbed) 12exp(−12λperturbedCsji2)∏s∈structures1(2πλR) 12exp(−12λRWjs2)∏s∈structures1(2πλΔ) 12exp(−12λΔ(|WWTs−Wjs|−|WWT0,s−W0,js|)2) (12)

Corresponding to a log-posterior (obviating the normalizing factor):

logp(W,Ψ,D,C|Dobs)=−nm2log(2π)+∑ i∈positionsj∈measurements−log(Ψi)2−12Ψi(Djiobs−∑s∈structuresWjs(Dsi+Csji))2+∑s∈structureslog(λsi)−λsiDsi+∑i∈perturbed(s,j)−12log(2πλperturbed)−12λperturbedCsji2+∑s∈structures−12log(2πλR)−12λRWjs2+∑s∈structures−12log(2πλΔ)−12λΔ(|WWTs−Wjs|−|WWT0,s−W0,js|)2 (13)

For the E-step, we estimate optimal hidden variable values for *D* and *C*. For each position *i*, finding the hidden variable for each structure *s*, *D*_*si*_ that maximizes log *p* by differentiating and setting to zero, gives the following linear equations:

$$\begin{array}{c} \sum_{\begin{array}{l} j\in \text{measurements}\\ s\prime \in \text{structures} \end{array}}W_{js}W_{js\prime }\left(D_{s\prime i}+C_{s\prime ji}\right)=\sum_{j\in \text{measurements}}W_{js}D_{ji}^{obs}+\lambda_{si}\Psi_{i},\\ \forall s\in \text{structures} \end{array}$$ (14)

For measurement *j*, if position *i* lies in *perturbed(s*,*j)*, solving for *C*_*sji*_ in the same manner gives the equation:

$$\begin{array}{c} \sum_{s\prime \in \text{structures}}W_{js}W_{js\prime }\left(D_{s\prime i}+C_{s\prime ji}\right)-\Psi_{i}\lambda_{perturbed}C_{sji}=W_{js}D_{ji}^{obs}\\ \forall s\in \text{structures}\\ \forall j\in \text{measurements} \end{array}$$ (15)

For each position *i*, compiling the Eqs (14) and (15) for all structures and measurements results in a linear system. Solving these *n* systems (one per sequence position), we obtain optimal values for *D*, *C*, which we name D*, C*.

For the M-step, we calculate *W* by maximizing log *p* in each measurement enforcing the probability distribution constraints in Eq (2). We can cast this maximization as a set of quadratic programs. Let $D_{j}^{obs}$ be the data and *W*_*j*_ be the set of weights for measurement *j*, then, to estimate the optimal weights for *j*, we solve:

$$\begin{array}{c} \underset{W_{j}}{\text{argmax}}\text{ log}p\left(W_{j},\;\Psi \text{,}D^{*},C^{*}|D_{j}^{obs}\right)\\ \text{subject to }\overset{t}{\underset{s\in \text{structures}}{\sum }}W_{js}=1\text{ and }W_{js}\geq 0 \end{array}$$ (16)

We solve the resulting quadratic programs using the CVXOPT python library [54] and denote the resulting weight matrix estimate as W*. Re-estimation of the variances Ψ_*i*_ in the M-step is also given by optimizing log *p*, but over Ψ_*i*_:

$$\Psi_{i}^{*}=\frac{1}{m}\sum_{j\in \text{measurements}}{\left(D_{ji}^{obs}-\sum_{s\in \text{structures}}W_{js}^{*}\left(D_{sj}^{*}+C_{sji}^{*}\right)\right)}^{2}$$ (17)

The E and M step optimization procedures are then repeated until the maximum difference of the induced base-pair probability matrices of the previous and current iteration is less than 1%. In our benchmarks, we have observed that usually 10 to 20 EM iterations are required for convergence. We note that our log posterior function is not convex and therefore our EM procedure does not necessarily converge to a global optimum and is sensitive to initial conditions. Nevertheless, testing different initial conditions with RNAstructure and ViennaRNA gave important improvements and similar results in our *in silico* benchmark (see Supporting Results in S1 Text above).

To initialize the variables Ψ_*i*_, we choose the empirical variance of position *i* across all chemical mapping measurements, consistent with the variance calculation performed when using M^2^ z-scores as pseudo-energy bonuses for secondary structure prediction [28]. For initial estimates of *W*, we use RNAstructure to calculate the energies of each structure in each mutant as in Eq (9).

To calculate uncertainties for *W* that are robust to outliers and high-reactivity values, we re-fit the model in bootstrapped datasets by sampling columns (i.e. sequence-positions) with replacement of *D* ^*obs*^ per bootstrap iteration. The final estimate of *W* is then the average weight matrix of all of the replicates; in all fits shown we report uncertainties as bootstrap standard deviations. Throughout this work, we used 100 bootstrap iterations. In the absence of bootstrapping, REEFFIT can also provide error estimates based on the Fisher information matrix approach, but those values are generally underestimates of the uncertainties.

### Building the structural ensemble and reducing model complexity

In most realistic scenarios for *de novo* RNA landscape modeling, it is not known *a priori* what set of structures would best model the data. To select an initial set of structures, we obtain a set of suboptimal structures for each sequence in the multi-dimensional chemical mapping experiment: for M^2^ experiments, the suboptimal structures of all variants involved have to be taken into account. We obtain at most 200 suboptimal structures of each mutant’s structural ensemble using the AllSub program, with default parameters (5% maximum energy difference from the MFE structure) from the RNAstructure program suite (version 5.5) [48].

The fits presented herein use all of the structures sampled in this manner. To reduce model complexity, we collapsed position-wise hidden reactivity variables that corresponded to different structures but formed part of the same structural motif. For example, if for position *i* we have 100 hidden reactivity variables that either correspond to a particular helix or an interior loop in the same part of the sequence, then we collapse the hidden variables into two variables, a helix and an interior loop variable, rather than 100 hidden reactivity variables. This variable collapse encodes the assumption that identical structural motifs will exhibit similar chemical reactivities independent of the structural context.

Specifically, let *sm* be a structural motif (the structural motifs that we take into account are: helices, bulges, *x*-way junctions, interior loops, dangles, single-stranded regions between helices, and hairpin loops) and let *motif(i*, *sm)* be the structures that have the *sm* motif at position *i*. Then, we constrain the values for all variables in the set {*D*_*si*_|∀*s* ∈ *motif*(*i*,*sm*)}to be the same, collapsing the |*motif*(*i*,*sm*)| variables into only one variable. Because the number of structures sampled typically exceeds the number of measurements (in most cases analyzed here we obtained ensembles of over 200 structures), this model simplification is essential to prevent over-fitting. In cases where the same sub-motif may have different reactivities in different structures (e.g. upon ligand-binding), this information can be used to expand the number of fit parameters for that motif, as we carried out for the *add* or Tebowned riboswitches (see below).

To select the regularization parameters *λ*_Δ_ and *λ*_*R*_ we used a cross-validation approach. Since chemical mapping data are structured (i.e. we cannot assume that reactivities in all positions come from the same population given the structural context of each nucleotide), we have to adapt the cross-validation technique accordingly. We employed a strategy frequently used in structured data contexts such as spline smoothing, where the *i*-th subsample in a *k*-fold cross-validation is the sequence {*i*,*i*+*k*,*i*+2*k*,…}[55]. This strategy samples the structured data uniformly across the nucleotide sequence, maintaining the assumptions necessary for cross-validation. In each fold of the cross validation, we obtain structure weights using the data from the training samples. With these weight estimates, we then obtain best predictions of the positions in the test sets with these weights. The cross-validation error is then the mean square error of the predicted and the observed test data [56]. We calculated an optimal value for *λ*_Δ_ and *λ*_*R*_ using our *in silico*, *ab initio* benchmark of 20 Rfam members, minimizing the 10-fold cross-validation error across all the benchmark. The optimal values, *λ*_Δ_ = 5 and *λ*_*R*_ = 0.26 were then used for all fits presented herein.

## Data set and software availability

The REEFFIT programs and their source code are available at http://rmdb.stanford.edu/tools/docs/reeffit/, along with software documentation and tutorials. M^2^ capillary electrophoresis data for the Bistable, *add* riboswitch, M-stable, and Tebowned RNAs have been deposited in the RMDB (RMDB IDs BSTHPN_1M7_0000.rdat, ADDSCHW_1M7_0000, ADDSCHW_1M7_0001, MSTBL_1M7_0000, TBWND_1M7_0000, and TBWND_1M7_0001). M^2^-seq data for the MedLoop are part of the EteRNA cloud lab, rounds 72 (RMDB ID ETERNA_R72_0000, project name “MedLoop”). RDAT files for the simulated datasets can be downloaded from http://purl.stanford.edu/zr287dq2666

## Supporting Information

S1 Text Supporting Results, Methods, Figures, and Tables. (PDF)

S2 Text A Supporting Report with detailed figures on the *in silico* benchmark results. (PDF)
