# Correction: Rich RNA Structure Landscapes Revealed by Mutate-and-Map Analysis

**DOI:** 10.1371/journal.pcbi.1004783

**Published:** 2016-02-18

**Authors:** The PLOS Computational Biology Staff

The following errors were introduced during the production process:

- The first sentence of the Abstract is incomplete, and should read as follows:

“Landscapes exhibiting multiple secondary structures arise in natural RNA molecules that modulate gene expression, protein synthesis, and viral infection.”

- In the seventh line of the legend for Figure 1, “pro?le” should appear as “profile”.
- In the first line of the legend for Figure 6, “M2 data” should appear as “M^2^ data”.

The publisher apologizes for these errors.
