# Supplementary material for: Rich RNA Structure Landscapes Revealed by Mutate-and-Map Analysis
Source: PLoS Comput Biol. 2015 Nov 13;11(11):e1004473. doi: 10.1371/journal.pcbi.1004473 (PMC4643908; doi:10.1371/journal.pcbi.1004473)
Supplement: S2 Text — (PDF) [file pcbi.1004473.s002.pdf]

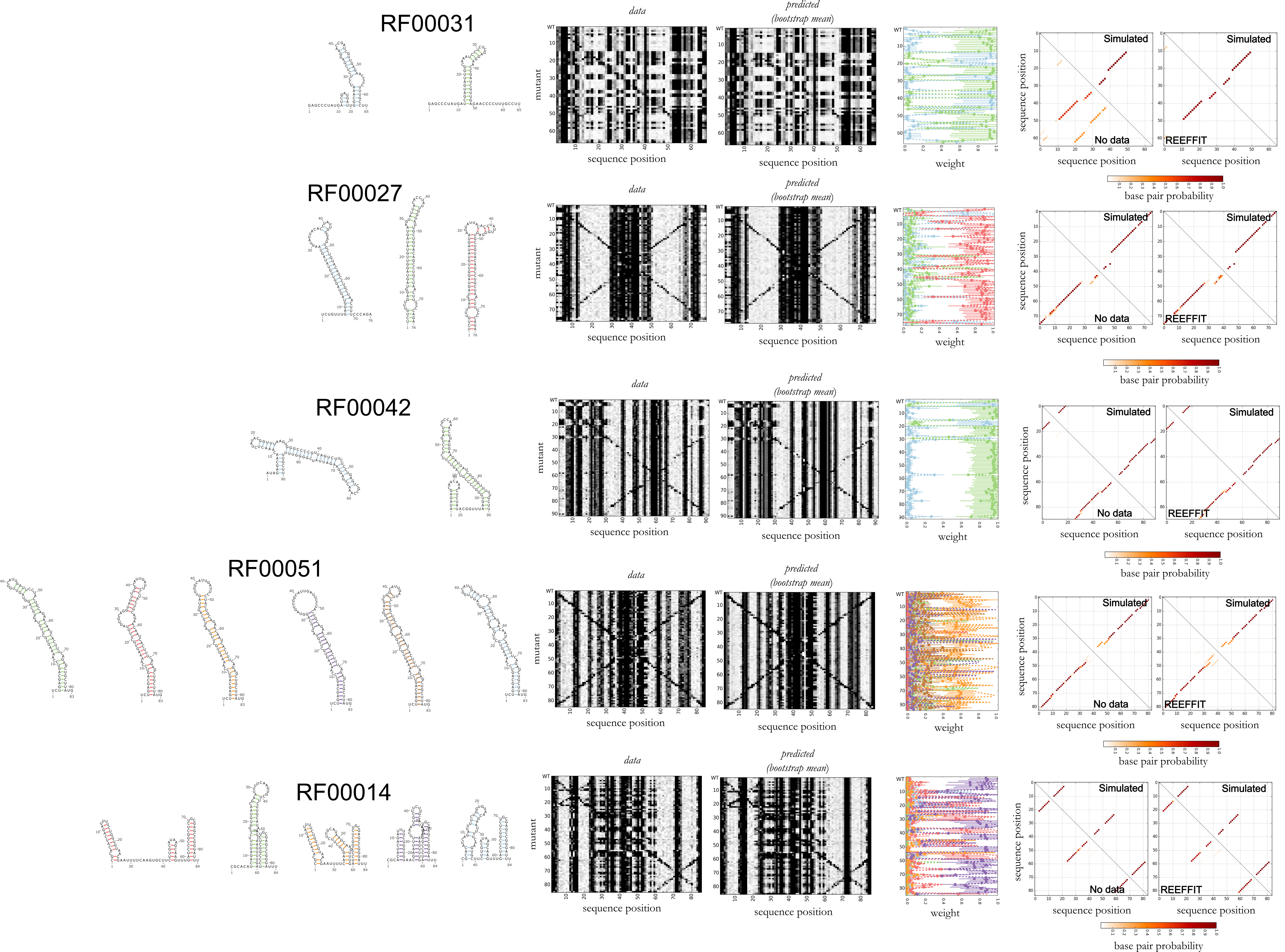
RF00031
RF00027
RF00042
RF00051
RF00014
data
predicted
(bootstrap mean)
mutant
WT
sequence position
weight
Simulated
No data
REEFFIT
base pair probability

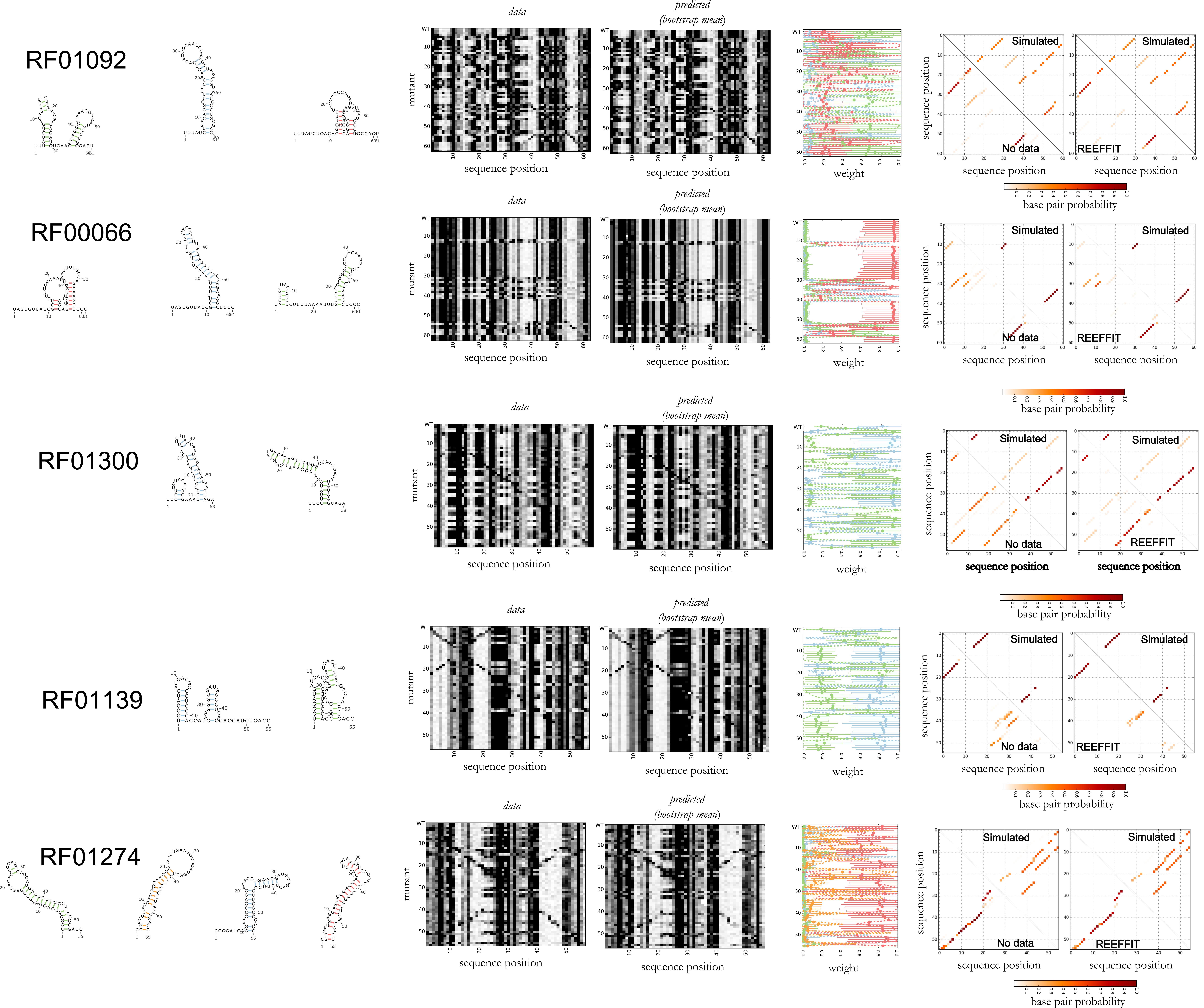
RF01092
RF00066
RF01300
RF01139
RF01274
data
predicted (bootstrap mean)
mutant
WT
sequence position
weight
Simulated
No data
REEFFIT
base pair probability

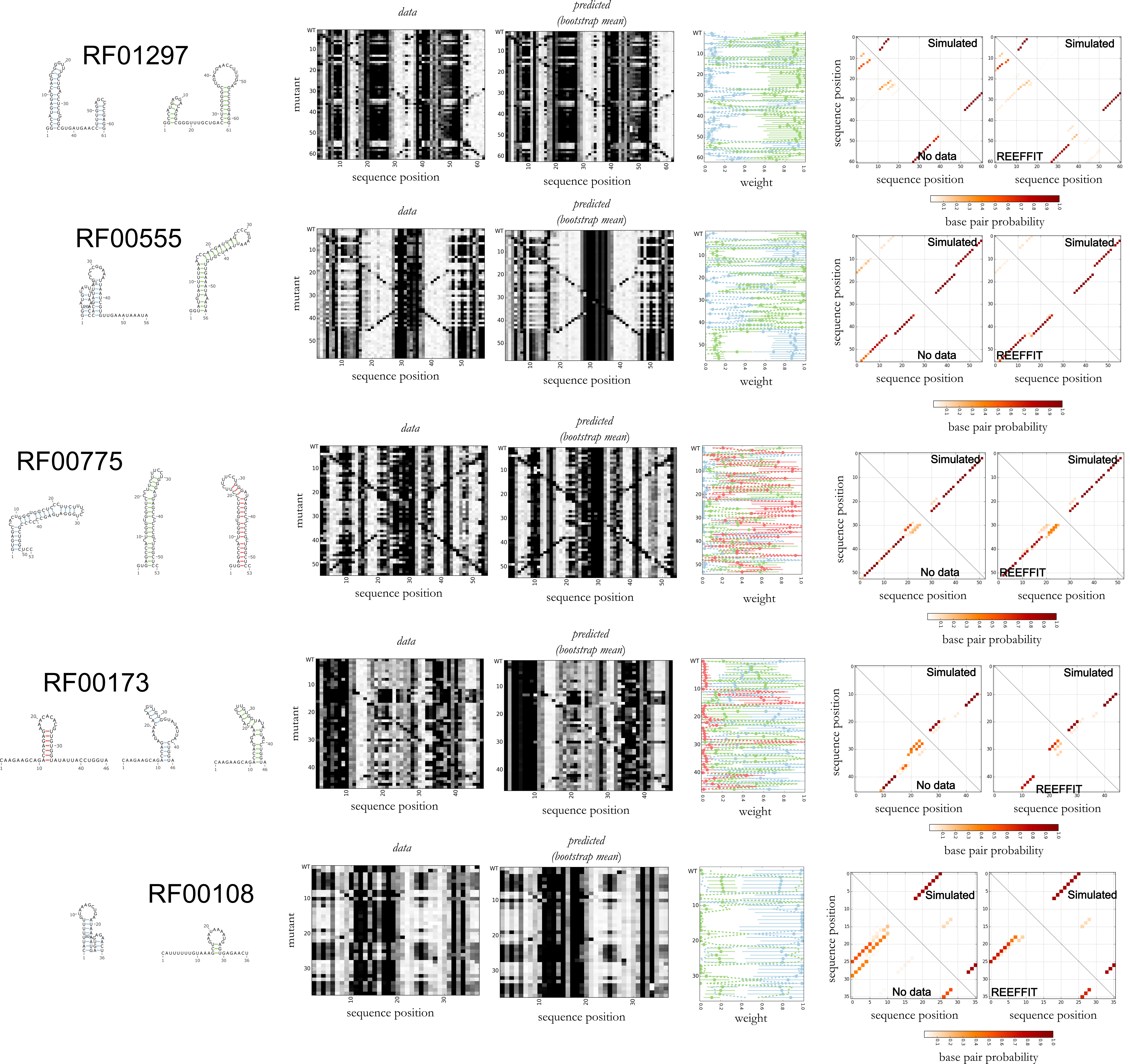
RF01297
RF00555
RF00775
RF00173
RF00108
data
predicted
(bootstrap mean)
mutant
WT
sequence position
weight
Simulated
No data
REEFFIT
base pair probability

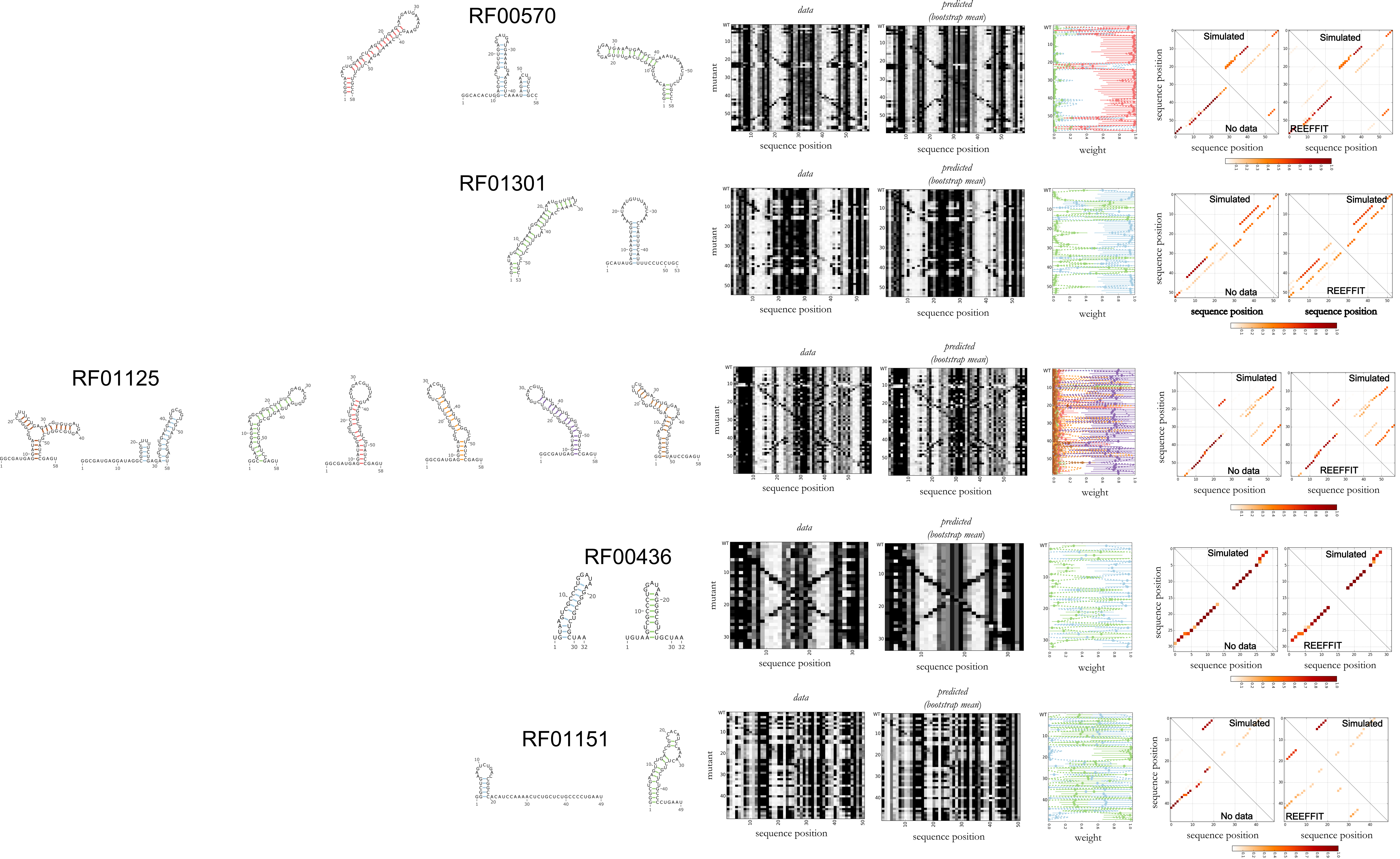
RF00570
RF01301
RF01125
RF00436
RF01151
data
predicted
(bootstrap mean)
mutant
sequence position
weight
Simulated
No data
REEFFIT
