# Supplementary material for: Rich RNA Structure Landscapes Revealed by Mutate-and-Map Analysis
Source: PLoS Comput Biol. 2015 Nov 13;11(11):e1004473. doi: 10.1371/journal.pcbi.1004473 (PMC4643908; doi:10.1371/journal.pcbi.1004473)

Supporting information for “Rich RNA structure landscapes revealed by mutate-and-map analysis”

Pablo Cordero[1,2] and Rhiju Das[1,2,3,*]

Affiliations:

[1]Biomedical Informatics Program, Stanford University, Stanford, CA 94305, USA

[2]Biochemistry Department, Stanford University, Stanford, CA 94305, USA

[3]Physics Department, Stanford University, Stanford, CA 94305, USA

*Correspondence to: Rhiju Das (rhiju@stanford.edu, 650-723-5976)

This document contains the following material:

- Supporting Results
- Supporting Methods
- Supporting References
- Supporting Tables
- Supporting Figures

Supporting Results

Quantitative landscape evaluation

Beyond its use as a tool for detecting dominant and alternative helices, we assessed REEFFIT's ability to quantitatively recover their populations in the *in silico* benchmark. The root-mean-squared deviation (RMSD) of fitted vs. actual helix probability over helices formed with at least 1% frequency was 10%. This accuracy is somewhat better than the mean precision over these helix frequencies estimated from non-parametric bootstrapping (15%, see Supporting Methods and Supporting Table E), supporting the use of bootstrapping as a conservative estimate of error on a helix-by-helix basis.

Example test cases with different landscape complexities in the *in silico* benchmark

Our simulated benchmark for landscape modeling included RNAs that folded into predominantly one structure, RNAs with excited states present at more than 10% population, and RNAs with complex landscapes in which various competing helices were present. Below we describe examples of each of these cases.

Supporting Figures Aa-e show simulated data for the sequence RF01301, the small nucleolar RNA SNOR4A. The benchmark simulation of this RNA involved 25 suboptimal secondary structures, most of them related by register-shifts to either a 10 nucleotide hairpin, 1301-A, or a more complex structure 1301-B comprised of 5 helices separated by internal loops (Supporting Figure Aa). The REEFFIT model provided an excellent fit to this RNA's simulated M^2 data; this is visually apparent in a side-by-side comparison (Supporting Figure Ab) and reflected quantitatively in a χ^2 /dof value of 1.09, close to the optimal value of 1.0. Recovered features included the 'diagonal' feature corresponding to disruptions near the site of each mutation as well as more dramatic features due to stabilization of state 1301-B upon certain mutations, visible as 'plaid' patterns in the simulated data (see, e.g. U14A, A41U). The importance of using a full ensemble fit was tested by repeating REEFFIT with the constraint that only 1, 2, or 3 structures were used in describing the data; these fits did not adequately capture all features in the simulated M^2 data (Supporting Figure D).

Beyond the quality of the fit, the actual details of the ensemble were recovered accurately, despite a substantial perturbation of states' population fractions from their original RNAstructure values. The improvement can be visualized in the matrices of base pair probabilities from RNAstructure and from REEFFIT compared to the actual simulated matrix (Supporting Figure Ac and Ad). Quantitatively, the total weights for 1301-A and 1301-B in the wild type sequence, simulated to be 58% and 42%, respectively, were recovered as helix fractions of 62±12% and 38±11%, respectively. Analogous accuracy was observed across all the simulated mutants (Figure Ae). Having the full mutate-and-map data set was important for making these inferences; using the wild type data alone (as would be carried out in conventional 'one-dimensional' chemical mapping[26]) returns a highly ambiguous fit with incorrect helix frequencies (see Supporting Figure E).

Supporting Figures Ae-h illustrate REEFFIT analyses for two other benchmark RNAs with qualitatively distinct ensembles. Tests on simulated data for the selenocysteine insertion sequence (SECIS, Rfam id RF00031, Figs. Af-h) supported the ability of M^2-REEFFIT to model an RNA with a single dominant secondary structure (95% population, recovered as 91±15%) without giving false positives for alternative structures present at low population. For complex cases, such as RF01300, REEFFIT was able to detect the 190 helices present at more than 25% population in the multiple states of the simulated ensembles of the wild type and 58 mutants (Fig. Ai-l). In both of these examples, analysis of the data for the wild type sequence alone gave poor recovery of the simulated ensembles (see Supporting Figure E), confirming the need for the full M^2 measurements.

Although in all the benchmark cases REEFFIT improved sensitivity and specificity to alternative helices, in some cases recovery of these hidden states was poor. Such was the case of RF00027, the let-7 miRNA, which contained three predominant structures in the ensemble: 27-A, 27-B, and 27-C (see Supporting Figures in S2 Text). Of these structures, which shared a long helix, only 27-A was present at more than 2% in the wild type sequence. The suboptimal ensemble of structures sampled for this RNA was a heterogeneous set of 1084 structures. Using these suboptimal structures, RNAstructure overestimated the wild type fraction of the helices associated with 27-C by 20%. REEFFIT did not correct this overestimation, but rather increased the fraction of helices associated with 27-B by 10%. This behavior was reflected throughout the mutants: the mean helix-wise false positives across mutants increased from 1.69 to 1.88 (see Table 1). Nevertheless, clustering the 1084 structures taking 27-A, 27-B, and 27-C as medoids revealed that the cluster weights were in agreement, within error, with the simulated weights in the wild type and across most mutants; for example, the cluster weight for 27-A, a bootstrapped mean, was lower, but within bootstrap error, than the respective simulated weight in the wild type (88 ± 15 % compared to 98%). Further, the model fit recovered most features of the simulated data, with an excellent χ^2 /dof of 1.03. This analysis indicates that the model given by REEFFIT is not necessarily worse than the prediction with no data, but rather an assessment of the uncertainties of the weights given the data.

Another case where REEFFIT failed to give an accurate landscape model of the wild type sequence was challenging multi-state RNA given by RF01092. This RNA's simulated ensemble was clustered in three states: 1092A, 1092B, and 1092C, which had almost no helices in common (see Supporting Figures in S2 Text). 1092-A had the highest fraction in the wild type (43%), followed by 1092-B (41%), and 1092-C (16%). We modeled this dataset with 96 suboptimal structures. The unperturbed RNAstructure predictions gave a mean helix weight of 75% to 1092-B, far from the true fraction of 42% but REEFFIT increased this value to 81%. However, when clustering the 96 suboptimal structures into three states to compare to the simulating structures, we found that REEFFIT predicted the weights of most mutants almost exactly and the mutant-wise HP-RMSD saw a substantial improved from the unperturbed RNAstructure estimates (18% RMSD for the unperturbed values vs. 12% RMSD for the REEFFIT model and a mean true positive helix recovery increase across mutants from 2.6 to 3.26, see Table 1 and Supporting Table E). The comparatively low improvement for the WT (no improvement in helix-wise TP and FP and HP-RMSD) was explained by the difference in the magnitude of the simulated perturbations for the wild type and the rest of the mutants (an average of 1.1 kcal/mol for the mutants and 4.9 kcal/mol for the wild type). This difference was in disagreement with one of our strategies for regularization, which penalizes solutions that deviate too much from $\Delta\Delta G$ values between wild type and

mutant structure fractions (see Materials and Methods). The situation, where there is a drastic difference between the prediction errors for the wild type and the mutants, was possible, albeit rare, in our simulations, since we generated the perturbations randomly and independently for each mutant; however, this situation might be unexpected in real systems.

Evaluation using ViennaRNA

As a further systematic check, we evaluated REEFFIT using an alternative secondary structure package, ViennaRNA [30]. Initial structures and population fractions predicted by ViennaRNA without M^2 data gave high FNR (20%) and FDR of (26%). Incorporation of the M^2 data by seeding REEFFIT with these structures and initial population fractions gave a significant improvement in FDR (11%), but only a modest improvement in FNR (18%; see Supporting Table F). In terms of population fractions, the ViennaRNA models gave similar helix frequency RMSDs (12% and see Supporting Table G) comparable to RNAstructure values.

Including compensatory mutation measurements in REEFFIT analysis of the 126-235 16S domain

REEFFIT analysis of the 16SFWJ M^2 data data suggested weak populations of alt-P1d (26±18%) and alt-P4 (18±10%), albeit with high errors estimated by bootstrapping. These results convey the uncertainty of the M^2 data in the alt-P1d, alt-P4, and P4 regions, similar to our expert analysis prior to compensatory rescue experiments. We therefore tested if REEFFIT could refine its inferences upon inclusion of data for compensatory mutations designed to test different helices and previously interpreted manually. Indeed, the likelihood of helices alt-P1d and alt-P4, for which no compensatory rescue evidence was found, was reduced, as the estimated errors on their population fraction increased (both with weights 24±21%, signal to noise ratio of 1.1, at our detection threshold). Other helices for which compensatory mutants were observed to give rescue (P4a and shiftP4a) remained, but now with higher confidence and reduced uncertainties (28±11% and 67±14%, see Figure E).

FMN titrations of mutants stabilizing TBWN-A/B/C

While we were able to test REEFFIT predicted reactivities by stabilizing each state of the Tebowned switch, an additional set of tests of the REEFFIT model was enabled by the inclusion of the FMN aptamer sequence in these RNAs. We tested predictions that the state-isolating mutants would show improved (for TBWN-B) or worsened (for TBWN-A & TBWN-C) affinities for the FMN ligand compared to the wild type sequence. FMN binding experiments monitored by dimethyl sulfate mapping [33] confirmed these predictions, with the TBWN-B-stabilizing mutants giving dissociation constants of K_d = 2–5 and 3–29 μM, improved by a ratio of 8 to 250-fold compared to the wild type RNA (250—530 μM). The mutants stabilizing TBWN-A and TBWN-C gave poorer binding than wild type (K_d of 3700 μM and > 5000 μM for TBWN-A mutants; and 3000 μM and > 5000 μM for TBWN-C mutants; Figure 5 and Supporting Figure H). Overall, both mutational and ligand binding analyses support the REEFFIT ensemble of at least three states in the Tebowned RNA, which in turn explain the structural discrepancies observed in initial characterization of this imperfectly designed riboswitch.

Supporting methods

Other strategies for ensemble (model) selection

We explored other approaches for selecting the structural ensemble to fit to the data. In particular, we tested to see if few small subsets of the sampled suboptimal structures gave better fits than most subsets. Theoretically, to find the best structures that model the data we could enumerate all possible combinations of structures from the suboptimal structures, fit our model to each combination, calculate a penalized information score, and choose the combination with the best score. In practice, this method is intractable for a large set of suboptimal structures due to the exponentially large number of possible combinations. Further, the quadratic nature of the likelihood function does not allow for a nested decomposition required for search space reduction techniques such as dynamic programming. Nevertheless, we performed MCMC on the combination of structures, using the Akaike information criterion (AIC) as a scoring function and adding and deleting structures as the Monte Carlo moves. Even for the simplest cases, such as the MedLoop RNA, several combinations of structures yielded similarly optimal AIC scores, indicating ambiguities when selecting a subset of structures. Furthermore, these AIC-equivalent combinations of structures modeled some parts of the dataset better than others. Other strategies that we tested, including a greedy "hill climbing" algorithm and using SHAPE-directed modeling between EM iterations to guide the structure selection procedure gave similar results, indicating potentially important missing variables in these limited models and suggesting that the best strategy would be to model as many relevant structures as possible – a global ensemble fit – with stringent regularization. This global ensemble fit captures many features that are missed when using a small set of structures, as can be seen in Figure C. When properly controlling for over-fitting, as with the bootstrapped uncertainties, weight regularization, and structural motif decomposition approaches outlined above, even models with a large number of structures could be fit to the data, returning conservative error estimates via bootstrapping, and making testable predictions.

Modeling the presence of a ligand

In two experimental cases, the add adenine riboswitch and the synthetic Tebowned FMN switch, we probed the RNAs in the presence of ligand (5 mM adenine and 2 mM FMN respectively). To model these data, we included copies of the aptamer-forming structures but changed the reactivity priors of the positions that are known to interact with the ligand from unprotected to protected. Further, for the data with ligand present, we gave ligand-protected structures a 20 kcal/mol bonus to simulate ligand binding and forced these structures to stay within 10 kcal/mol of their starting energies.

Visualizing the fitted ensemble

Visualizing the base pairing patterns and fractions of possibly hundreds of fitted structures in the wild type ensemble and across all mutants is a challenging data visualization problem. To achieve this, we employed two different strategies. First, to visualize changes from the REEFFIT-predicted ensemble from initial RNAstructure values, we plotted base pair probability matrices comparing the RNAstructure fractions to the final REEFFIT values (see, e.g., Figure 1). To show broad structural ensemble changes between variants, we grouped all of the fitted structures into a reduced number of states using hierarchical clustering with base pair distance as the metric. To set a cutoff for the dendrogram, we chose a threshold that maximizes the Calinski-Harabasz index that has been previously used to cluster RNA secondary structures [13]; the structure medioids of each cluster are then used as representatives

of each states. This automated clustering of the most populated structures into states are used to help interpret and visualize the modeled ensembles across variants – the state weights corresponding to the sum of the weights of structures for each cluster can be readily visualized across perturbations (see Figures 1, F and H). We emphasize that this clustering mechanism is for visualization purposes only and that predictions of experimental observables, such as base pair and helix probabilities (shown in Figures 1-6 and used as our evaluation criteria in all Tables) are independent of this clustering scheme.

Simulating M^2 data

We used the following procedure to simulate M^2 SHAPE data for a given sequence.

1. We start with the set of structures taken from all mutants whose energies were calculated to be at most 5% above the minimum free energy structure obtained by the AllSub RNAstructure program.
2. We then decomposed these structures into motifs (helices, bulges, n-way junctions, single-stranded regions, dangles, and hairpin loops).
3. We generated mock reactivity profiles for each motif by sampling reactivities $RMDB_U$ and $RMDB_P$ for unpaired and paired residues respectively.
4. Additionally, we added a term, chosen uniformly at random, to all nucleotides in the motif (from the [-0.1, 0.1] interval for helices and for the [-0.1, 0.5] interval for the rest of the motifs). This introduces correlations that are typically observed across sets of paired and unpaired regions in SHAPE data. This term is ignored for reactivities that would become negative when adding these correlations.
5. For each single-nucleotide mutant, we calculated the free energies of the structures given the mutated sequence using the ef2n RNAstructure program[48]. For the perturbed ensembles of the *ab initio* benchmark, we added Gaussian noise (mean of 0, standard deviation of 1 kcal/mol) to the energies simulate deviations arising from our incomplete understanding of RNA thermodynamics.
6. Using these noisy energies, we calculated the Boltzmann weights of each structure and for each mutant. The reactivity profile of each mutant was then the linear combination of the reactivity profiles of each structure weighted by the set of weights calculated previously.
7. To simulate local perturbations due to mutations, we randomly added Gaussian-distributed reactivity differences in sites at most one nucleotide away from the mutation position and any base pair affected by the nucleotide change.
8. We then simulated measurement errors. For each sequence position *i*, we sampled an error variance uniformly at random from the [0.01 , 1] interval and scaled it by a factor of one half of the mean reactivity value across mutants in *i,* in line with the empirical observation that higher reactivities typically have higher measurement errors. We then added zero-mean Gaussian errors to each position using these variances.

These simulated datasets showed strong visual similarity to experimental M^2 data, such as global structure rearrangements and punctate features marking concomitant release of base pairing nucleotides upon mutation of one partner (see Figure 1).

Evaluation metrics for *in silico* benchmark

To evaluate the performance of REEFFIT in the *in silico* benchmark, we used two validation strategies. First, we evaluated the ability of REEFFIT to detect alternative helices from M^2 data while at the same time avoiding false positives. For each helix with average base-pair fraction of at least 25%, we considered it a true positive (TP) if the predicted model included at least half of its base pairs with at least 25% fraction. Similarly we considered helices for which at least half of its base pairs were predicted to be present in at least 25% fraction to be false positive (FP) predictions if these helices were not present in at least 25% fraction in the simulated model. These metrics are presented in Tables 1 and A.

Second, to evaluate the quality of the fits, we used the χ^2/dof statistic to assess goodness of fit and an average helix-wise root mean square deviation (HP-RMSD), either in the wild-type or across variants. Let HP_{js}^{ref} and HP_{js}^{pred} be true and predicted helix-wise probabilities for helix *j* in structure *s*, respectively (here, helix-wise probability is calculated as the mean of the base pair probabilities in that helix) and HI the set of nucleotides whose helix probability is greater than 1% in either the reference or the prediction. The HP-RMSD is then:

$$\text{HP-RMSD}\left(HP^{ref}, HP^{pred}\right) = \sqrt{\frac{\sum_{j,s \in HI}\left(HP_{js}^{ref} - HP_{js}^{pred}\right)^2}{|HI|}} \qquad (17)$$

The restriction of the HP-RMSD to pairs with probabilities greater than 1% reduces biases towards low HP-RMSDs for large RNA sequences. These metrics are presented throughout the supporting tables.

Data set and software availability

The REEFFIT programs and their source code are available at http://rmdb.stanford.edu/tools/docs/reeffit/, along with software documentation and tutorials.

M^2 capillary electrophoresis data for the Bistable, *add* riboswitch, M-stable, and Tebowned RNAs have been deposited in the RMDB (RMDB IDs BSTHPN_1M7_0000.rdat, ADDSCHW_1M7_0000, ADDSCHW_1M7_0001, MSTBL_1M7_0000, TBWND_1M7_0000, and TBWND_1M7_0001).

M^2-seq data for the MedLoop are part of the EteRNA cloud lab, rounds 72 (RMDB ID ETERNA_R72_0000, project name “MedLoop”).

RDAT files for the simulated datasets can be downloaded from http://purl.stanford.edu/zr287dq2666

Supporting Tables

Table A: Helix-wise performance results for M^2-REEFFIT landscape dissection on a 20 RNA benchmark from the Rfam database across all simulated mutants.

Name	Sequence Length	Number of structures	χ^2/dof	Number of Helices [a]	No data TP	No data FP	REEFFIT TP	REEFFIT FP
RF00031 SECIS	65	85	1.01	177	133	76	162	1
RF00051 mir-17	83	652	1.02	429	407	16	420	13
RF00014 DsrA	84	594	0.96	338	300	49	327	7
RF01092 GP_knot2	61	96	1.01	184	125	61	179	25
RF00066 U7	61	77	1.01	177	96	96	141	69
RF01300 snoU49	58	64	0.94	190	142	85	179	4
RF01139 sR2	55	124	0.98	136	114	57	127	1
RF01274 sR45	55	195	1.10	123	105	19	111	35
RF01297 sR40	61	511	1.04	184	125	100	170	10
RF00555 L13_leader	56	64	1.05	161	134	30	157	4
RF00775 mir-432	53	137	1.11	250	189	28	231	34
RF00173 Hairpin	46	43	1.09	103	85	41	96	21
RF00108 SNORD116	36	25	1.03	64	39	68	46	56
RF00570 SNORD64	58	102	1.10	194	148	30	180	16
RF01301 snoR4a	53	59	1.00	131	82	90	121	39
RF01125 sR4	58	140	1.02	139	117	22	130	8
RF00436 UnaL2	32	32	1.06	57	57	10	57	0
RF01151 snoU82P	49	59	1.04	79	49	50	76	19
RF00027 let-7	76	1084	1.03	243	235	28	241	26
RF00042 CopA	90	1069	1.06	554	528	21	549	10
				**FDR**	23.3%		9.7%	
				**FNR**	18%		5.4%	

[a] Helices are a minimum 3 base pairs long and are present in a minimum 25% population fraction.

Table B: Base-pair-wise performance of M^2-REEFFIT in the *in silico* Rfam benchmark across all mutants

Name	Sequence Length	Number of structures	χ^2/dof	Number of Base pairs	No data TP	No data FP	REEFFIT TP	REEFFIT FP
RF00031 SECIS	65	85	1.01	1179	936	548	1119	37
RF00051 mir-17	83	652	1.02	2372	2252	193	2310	155
RF00014 DsrA	84	594	0.96	2544	2252	443	2438	51
RF01092 GP_knot2	61	96	1.01	994	592	357	878	175
RF00066 U7	61	77	1.01	781	477	496	640	380
RF01300 snoU49	58	64	0.94	1143	824	520	1031	78
RF01139 sR2	55	124	0.98	758	600	370	643	56
RF01274 sR45	55	195	1.10	1046	878	309	928	247
RF01297 sR40	61	511	1.04	1108	786	507	1031	116
RF00555 L13_leader	56	64	1.05	1066	922	203	1040	52
RF00775 mir-432	53	137	1.11	1141	865	127	1057	161
RF00173 Hairpin	46	43	1.09	551	444	233	460	94
RF00108 SNORD116	36	25	1.03	307	177	347	235	239
RF00570 SNORD64	58	102	1.10	1126	883	324	1054	116
RF01301 snoR4a	53	59	1.00	848	589	377	792	150
RF01125 sR4	58	140	1.02	913	815	256	874	180
RF00436 UnaL2	32	32	1.06	321	318	59	320	43
RF01151 snoU82P	49	59	1.04	443	279	340	404	181
RF00027 let-7	76	1084	1.03	2122	2042	251	2094	227
RF00042 CopA	90	1069	1.06	2873	2716	242	2833	152
				**FDR**	24.9%		11.5%	
				**FNR**	16.9%		6.2%	

Table C: Performance of M^2-REEFFIT in the *in silico* Rfam benchmark for the wild type sequence alone

Name	Sequence Length	Number of structures	χ^2/dof	Number of Helices [a]	No data TP	No data FP	REEFFIT TP	REEFFIT FP
RF00031 SECIS	65	85	1.01	9	2	2	2	0
RF00051 mir-17	83	652	1.02	17	5	0	6	0
RF00014 DsrA	84	594	0.96	7	4	0	4	0
RF01092 GP_knot2	61	96	1.01	11	3	0	3	0
RF00066 U7	61	77	1.01	3	2	1	2	1
RF01300 snoU49	58	64	0.94	13	3	2	3	1
RF01139 sR2	55	124	0.98	4	2	1	2	0
RF01274 sR45	55	195	1.10	13	2	0	2	1
RF01297 sR40	61	511	1.04	15	2	1	2	0
RF00555 L13_leader	56	64	1.05	6	3	0	3	0
RF00775 mir-432	53	137	1.11	13	3	1	4	1
RF00173 Hairpin	46	43	1.09	9	2	1	2	0
RF00108 SNORD116	36	25	1.03	4	2	2	2	0
RF00570 SNORD64	58	102	1.10	8	1	2	1	2
RF01301 snoR4a	53	59	1.00	3	2	1	5	0
RF01125 sR4	58	140	1.02	10	1	1	1	2
RF00436 UnaL2	32	32	1.06	8	2	0	2	0
RF01151 snoU82P	49	59	1.04	9	0	2	1	1
RF00027 let-7	76	1084	1.03	9	3	0	3	1
RF00042 CopA	90	1069	1.06	16	6	0	6	0
				**FDR**	25.4%		15.2%	
				**FNR**	24.2%		15.2%	

[a] Helices are a minimum 3 base pairs long and are present in a minimum 25% population fraction.

Table D: REEFFIT performance in the *in silico* Rfam benchmark using only one-dimensional chemical mapping (WT) data.

Name	Sequence Length	Number of structures	χ^2/dof	Number of Helices [a]	No data TP	No data FP	REEFFIT (1D data) TP	REEFFIT (1D data) FP
RF00031 SECIS	65	85	-0.09	9	2	2	2	2
RF00051 mir-17	83	652	1.02	17	6	5	0	5
RF00014 DsrA	84	594	0.97	7	4	4	0	4
RF01092 GP_knot2	61	96	-0.08	11	5	3	0	3
RF00066 U7	61	77	1.01	3	2	2	1	2
RF01300 snoU49	58	64	1.1	13	3	3	2	3
RF01139 sR2	55	124	0.94	4	2	2	1	2
RF01274 sR45	55	195	-0.04	13	4	2	0	2
RF01297 sR40	61	511	0.98	15	2	2	1	2
RF00555 L13_leader	56	64	1.04	6	3	3	0	3
RF00775 mir-432	53	137	1.05	13	4	3	1	3
RF00173 Hairpin	46	43	1.11	9	2	2	1	2
RF00108 SNORD116	36	25	1.09	4	2	2	2	2
RF00570 SNORD64	58	102	1.03	8	5	1	2	1
RF01301 snoR4a	53	59	1.03	3	5	2	1	2
RF01125 sR4	58	140	-0.05	10	3	1	1	1
RF00436 UnaL2	32	32	1.1	8	2	2	0	2
RF01151 snoU82P	49	59	1	9	1	0	2	1
RF00027 let-7	76	1084	1.01	9	3	3	0	3
RF00042 CopA	90	1069	0.96	16	6	6	0	6
				**FDR**	25.4%		25%	
				**FNR**	24.2%		22.7%	

[a] Helices are a minimum 3 base pairs long and are present in a minimum 25% population fraction.

Table E: M^2-REEFFIT performance in the *in silico* Rfam benchmark as measured by helix-wise RMSD.

					No data		REEFFIT	
Name	Sequence Length	Number of structures	χ^2/dof	Number of Helices [a]	WT HP-RMSD	All mutant HP-RMSD	WT HP-RMSD	All mutant HP-RMSD
RF00031 SECIS	65	85	1.01	9	14%	15%	5%	8%
RF00051 mir-17	83	652	1.02	17	10%	9%	5%	8%
RF00014 DsrA	84	594	0.96	7	2%	10%	5%	7%
RF01092 GP_knot2	61	96	1.01	11	18%	18%	17%	12%
RF00066 U7	61	77	1.01	3	12%	17%	8%	11%
RF01300 snoU49	58	64	0.94	13	16%	19%	14%	10%
RF01139 sR2	55	124	0.98	4	12%	12%	10%	10%
RF01274 sR45	55	195	1.10	13	19%	13%	18%	11%
RF01297 sR40	61	511	1.04	15	10%	16%	9%	10%
RF00555 L13_leader	56	64	1.05	6	10%	18%	6%	7%
RF00775 mir-432	53	137	1.11	13	20%	18%	15%	11%
RF00173 Hairpin	46	43	1.09	9	16%	17%	10%	13%
RF00108 SNORD116	36	25	1.03	4	24%	28%	12%	15%
RF00570 SNORD64	58	102	1.10	8	35%	16%	28%	10%
RF01301 snoR4a	53	59	1.00	3	18%	21%	7%	9%
RF01125 sR4	58	140	1.02	10	27%	11%	22%	8%
RF00436 UnaL2	32	32	1.06	8	6%	15%	4%	7%
RF01151 snoU82P	49	59	1.04	9	34%	23%	10%	12%
RF00027 let-7	76	1084	1.03	9	6%	9%	9%	8%
RF00042 CopA	90	1069	1.06	16	1%	5%	6%	6%
				**Average**	16%	16%	11%	10%

[a] Helices are a minimum 3 base pairs long and are present in a minimum 25% population fraction.

Table F: M^2-REEFFIT performance on all mutants in the *in silico* Rfam benchmark using ViennaRNA instead of RNAstructure to estimate initial energies.

					No data		REEFFIT (ViennaRNA)	
**Name**	**Sequence Length**	**Number of structures**	**χ^2/dof**	**Number of Helices [a]**	**TP**	**FP**	**TP**	**FP**
RF00031 SECIS	65	85	1.03	2.68	2.02	1.15	2.24	0.06
RF00051 mir-17	83	659	1.02	5.11	4.85	0.19	4.94	0.26
RF00014 DsrA	84	735	1.02	3.98	3.52	0.58	3.85	0.15
RF01092 GP_knot2	61	96	1.07	2.97	2.02	0.98	2.19	0.05
RF00066 U7	61	77	1.06	2.85	1.55	1.55	1.10	0.94
RF01300 snoU49	58	64	1.04	3.25	2.41	1.44	2.71	0.02
RF01139 sR2	55	124	1.04	2.43	2.04	1.02	1.95	0.16
RF01274 sR45	55	195	1.05	2.20	1.88	0.34	1.96	0.66
RF01297 sR40	61	543	1.02	2.97	2.02	1.61	2.50	0.26
RF00555 L13_leader	56	64	1.05	2.82	2.35	0.53	2.58	0.40
RF00775 mir-432	53	137	1.08	4.63	3.50	0.52	3.81	0.61
RF00173 Hairpin	46	43	1.11	2.19	1.81	0.87	1.68	0.36
RF00108 SNORD116	36	25	1.12	1.73	1.05	1.84	1.24	1.03
RF00570 SNORD64	58	102	1.03	3.29	2.51	0.51	2.90	0.25
RF01301 snoR4a	53	59	1.05	2.43	1.52	1.67	2.35	0.57
RF01125 sR4	58	140	1.07	2.41	1.98	0.37	2.29	0.12
RF00436 UnaL2	32	32	1.19	1.73	1.73	0.30	1.73	0.00
RF01151 snoU82P	49	59	1.05	1.66	0.98	1.00	1.46	0.28
RF00027 let-7	76	1111	1.02	3.16	2.96	0.34	3.30	1.80
RF00042 CopA	90	1069	1.01	6.09	5.80	0.23	6.03	0.11
				**FDR**	26%		11%	
				**FNR**	20%		18%	

[a] Helices are a minimum 3 base pairs long and are present in a minimum 25% population fraction.

Table G: M^2-REEFFIT performance in the *in silico* Rfam benchmark as measured by helix-wise RMSD using ViennaRNA.

					No data		REEFFIT (ViennaRNA)	
**Name**	**Sequence Length**	**Number of structures**	**χ^2/dof**	**Number of Helices [a]**	**WT HP-RMSD**	**All mutant HP-RMSD**	**WT HP-RMSD**	**All mutant HP-RMSD**
RF00031 SECIS	65	85	1.03	9	14%	15%	5%	8%
RF00051 mir-17	83	659	1.02	17	10%	9%	5%	9%
RF00014 DsrA	84	735	1.02	7	2%	10%	4%	6%
RF01092 GP_knot2	61	96	1.07	11	18%	18%	18%	11%
RF00066 U7	61	77	1.06	3	12%	17%	7%	9%
RF01300 snoU49	58	64	1.04	13	16%	19%	16%	15%
RF01139 sR2	55	124	1.04	4	12%	12%	9%	10%
RF01274 sR45	55	195	1.05	13	19%	13%	16%	12%
RF01297 sR40	61	543	1.02	15	10%	16%	7%	9%
RF00555 L13_leader	56	64	1.05	6	10%	18%	8%	10%
RF00775 mir-432	53	137	1.08	13	20%	18%	14%	14%
RF00173 Hairpin	46	43	1.11	9	16%	17%	16%	15%
RF00108 SNORD116	36	25	1.12	4	24%	28%	29%	18%
RF00570 SNORD64	58	102	1.03	8	35%	16%	26%	10%
RF01301 snoR4a	53	59	1.05	3	18%	21%	7%	9%
RF01125 sR4	58	140	1.07	10	27%	11%	22%	10%
RF00436 UnaL2	32	32	1.19	8	6%	15%	6%	8%
RF01151 snoU82P	49	59	1.05	9	34%	23%	4%	12%
RF00027 let-7	76	1111	1.02	9	6%	9%	13%	8%
RF00042 CopA	90	1069	1.01	16	1%	5%	7%	6%
				**Average**	16%	16%	12%	10%

[a] Helices are a minimum 3 base pairs long and are present in a minimum 25% population fraction.

Table H: Sequences experimentally probed with M^2-REEFFIT, written from 5´ to 3´. Sequences in blue represent the RNA domain of interest. Reference GAGUA (for M^2-CE) or barcode UUCG (for M^2-seq or MedLoop RNA) hairpins are highlighted in red.

Name	Sequence (numbering of region of interest)
MedLoop	GGAAAGGAACGACGAACCGAAAACCGAAGAAAUGAAGAAAGGUUUUCGGUACCGACCUGAAAACCUCUCACGUUCGCGUGAGAAAAAGAAACAACAACAACAAC (-9-50)
16S 126-235 domain	GGGAACGACUCGAGUAGAGUCGAAAAGGGAAACUGCCUGAUGGAGGGGGAUAACUACUGGAAACGGUAGCUAAUACCGCAUAACGUCGCAAGACCAAAGAGGGGGACCUUCGGGCCUCUUGCCAUCGGAUGUGCCCAAAGGAGUCGAGUAGACUCCAACAAAAGAAACAACAACAACAAC (126-235)
V. vulnificus add riboswitch	GGGAACCGAGUAGGUUCGCUUCAUAUAAUCCUAAUGAUAUGGUUUGGGAGUUUCUACCAAGAGCCUUAAACUCUUGAUUAUGAAGUCUGUCGCUUUAUCCGAAAUUUUAUAAAGAGAAGACUCAUGAAUGGGAUCCGAGUAGGAUCCUGAAAAAAACAACAACAAC (14-125)
Bistable	GGUGGCUUCGAGUAGAAGCCUUAUGUACCGGAAGGUGCGAAUCUUCCGAAGGAUCCGAGUAGGAUCCAAAAAGAAACAACAACAACAAC (1-25)
Tebowned	GGACGAUUGAGGAUAUUUAGACAUAGAGAAGGCAAUGGAGAGAGAGCUUUUUAAAGAAACAACAACAACAAC (1-52)
M-stable	GGAAACGCAACGAGUAGUUGCGUUACGCAGCAGUCCUCCAGAGGACAUUUUUCGAGCAGGUAGAGAGGAUCCGAGUAGGAUCCAAAGAAACAACAACAACAAC (1-44)

Table I: Folding conditions and statistics for experimental cases.

Name	Condition	Sequence length	Number of variants [a]	Number of suboptimal Structures	χ^2/dof	Number of helices in WT [a]	Average no. of helices in all variants*
MedLoop	50 mM Na-HEPES, 10mM $MgCl_2$ pH 8.0	60	61	230	1.17	2	1.8
16S 126-235 domain	50 mM Na-HEPES, 10mM $MgCl_2$ pH 8.0	110	111	1794	1.07	5	6.8
16S 126-235 domain M^2-rescue	50 mM Na-HEPES, 10mM $MgCl_2$ pH 8.0	110	215	3147	0.89	4	4.5
V. vulnificus add riboswitch	50 mM KCl, 25 mM K_3PO_4, 10mM $MgCl_2$ pH 6.5 (no adenine)	112	113	2043 [c]	1.27 [c]	6 [d]	5.8
V. vulnificus add riboswitch	50 mM KCl, 25 mM K_3PO_4, 10mM $MgCl_2$ pH 6.5 (5 mM adenine)	112	113	2043 [c]	1.27 [c]	8 [d]	12.1
Bistable	150 mM NaCl, pH 8.0	25	26	76	1.1	2	1.2
Tebowned	50 mM Na-HEPES, 10mM $MgCl_2$ pH 8.0 (no FMN)	54	49	27 [c]	1.24 [c]	2 [d]	1.8
Tebowned	50 mM Na-HEPES, 10mM $MgCl_2$ pH 8.0 (2mM FMN)	54	49	27 [c]	1.24 [c]	4 [d]	4.6
M-stable	50 mM Na-HEPES, 10mM $MgCl_2$ pH 8.0	44	45	91	1.5	1	1.4

[a] Includes the wild type sequence

[b] Helices are located in the sequence of interest region, are a minimum 3 base pairs long and are present in a minimum 25% population fraction.

[c] M^2 data in the presence and absence of ligand for the add and Tebowned RNA switches were modeled jointly by REEFFIT.

[d] Increase in number of helices upon ligand binding is due to suppression of ligand-bound state helices in ligand-free conditions, below 25% population fraction cutoff

Supporting Figures

Figure A: Three example cases of evaluating REEFFIT with simulated data. (a) Simulated M^2 data for the RF01301 snoR4a test case (b) Bootstrapped REEFFIt fits. (c) Base pair probability matrix comparisons between the simulated (true) base pair probabilities (upper half) and the unperturbed RNAstructure values; (d) analogous base pair probability comparison to M^2-REEFFIT estimates. (e) Cluster medioids of structures used for generating the M^2 simulated data. (f—j) Evaluation results for the RF00031 SECIS RNA simulated mapping data. (k—o) Evaluation results for the RF00014 DrsA RNA. Detailed figures on each of the benchmark cases are given in the Supporting Figures in S2 Text.

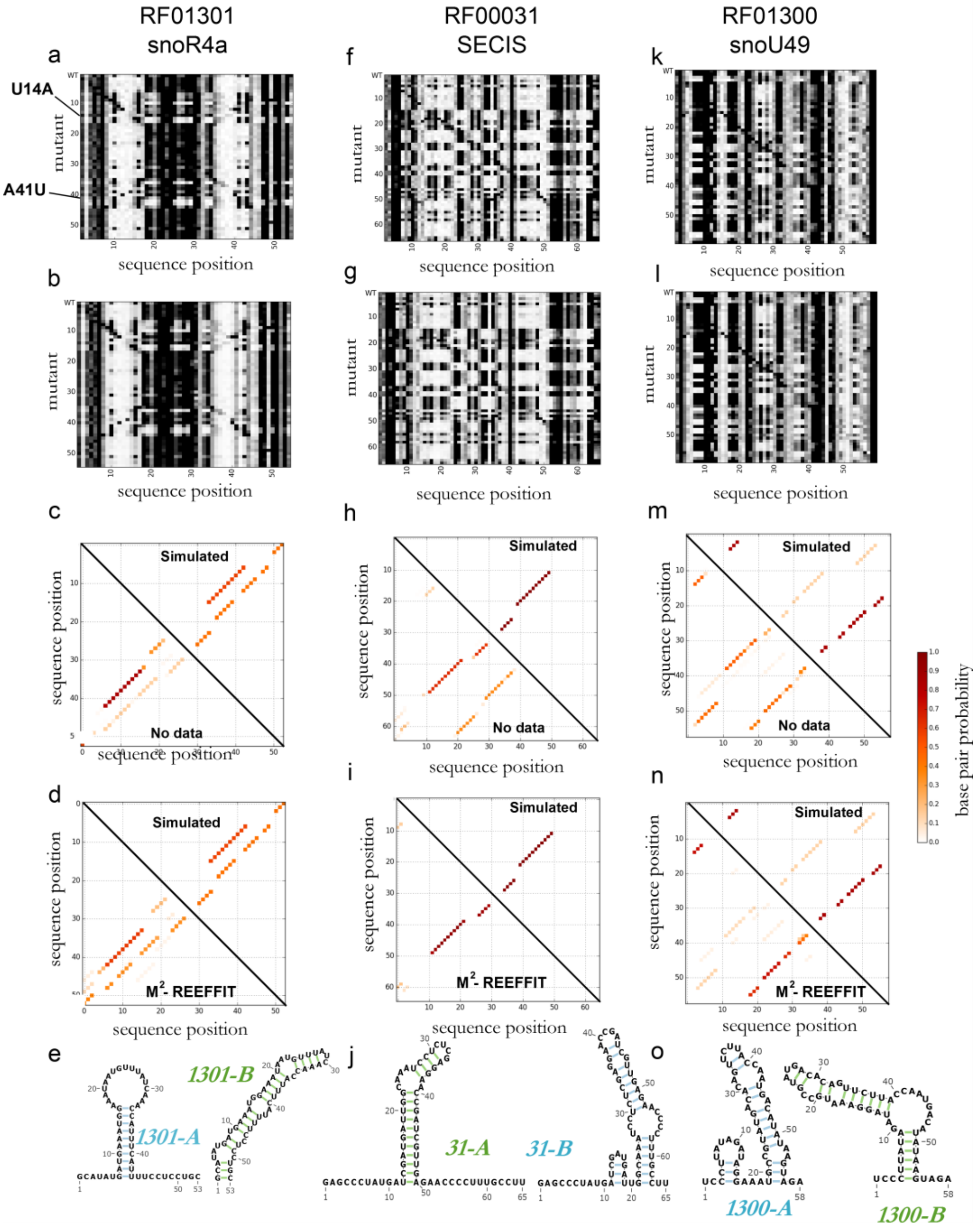

Figure B: Overall performance of REEFFIT for helix detection in the WT sequence of the simulated M^2 benchmark. (a) Precision-recall curve for various REEFFIT signal-to-noise thresholds for detecting helices present in at least 25% fraction in all probed variants. PPV = positive predictive value (false discovery rate, FDR = 1 – PPV). Blue dot: Reference FDR and FNR of RNAstructure baseline (no data). (b) Black: Precision-recall curve for various REEFFIT signal-to-noise thresholds for helix detection in the wild type sequence. Gray: analogous precision-recall curve when using only the WT (1D mapping) data. Blue dot: Reference FDR and FNR of RNAstructure baseline (no data).

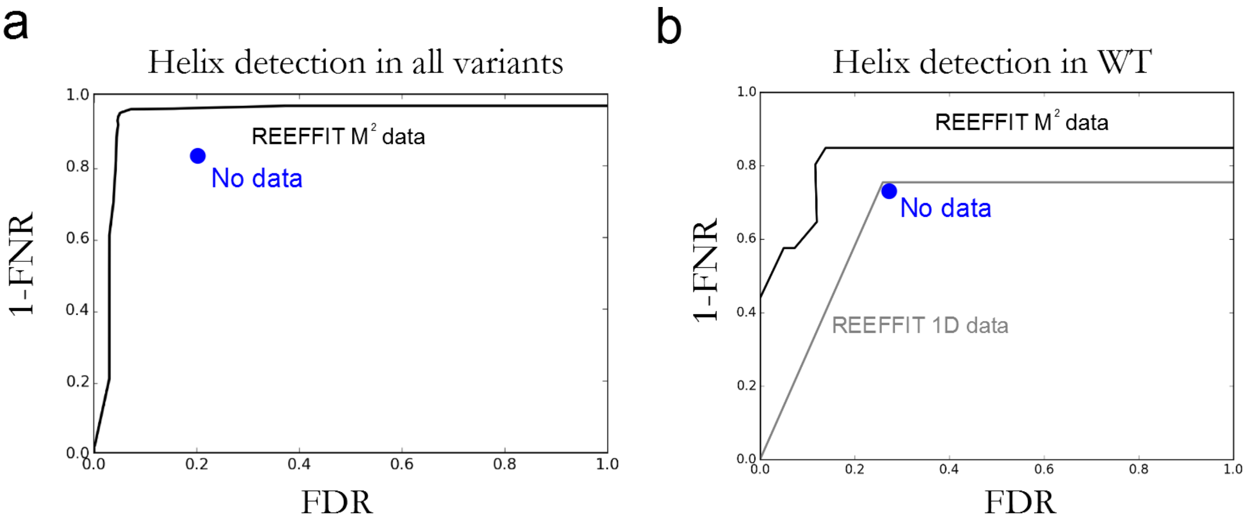

Figure C: Comparison of REEFFIT's global ensemble fit with few-state fits on the *in silico* benchmark test case RF01301 sno4a RNA. (a) Simulated M^2 data; (b) predicted data using a global ensemble fit; (c) 1-state, 2-state, and 3-state fits using top structures as scored by RNAstructure free energy. The fits (c) capture some, but not all, features seen in the simulated data when compared to a global ensemble fit. (d) Comparison of predicted data for the wild-type sequence by the 3-state and global ensemble models. Several features (e.g. positions 9, 15, and 42) are not adequately captured in the 3-state fit but are predicted by the global ensemble fit.

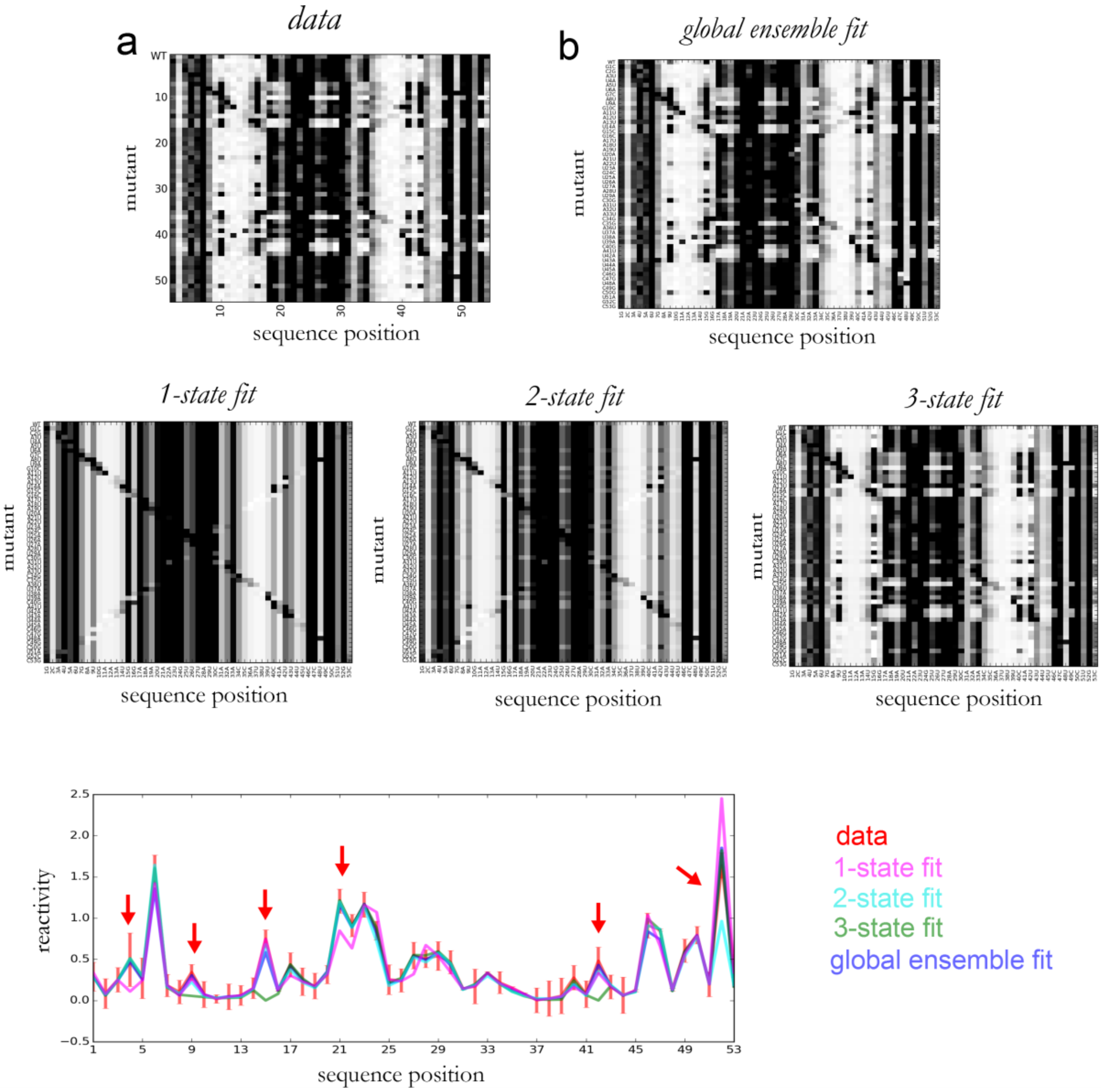

Figure D: Fitting only the wild type (one-dimensional) chemical mapping profile provides little information on the underlying ensemble. Base-pair probability matrix plots of the inferred landscapes using no data (a and d), only one-dimensional (b and e) and the full M^2 data (c and f) for the *in silico* test cases (a-c) RF01301 sboR4a and (d-f) RF00031 SECIS RNAs– each plot has the simulated, true, base-pair probability plot in the upper triangle for reference. In both cases, one-dimensional chemical mapping data fails to provide sufficient information to correctly model the folding landscape of these RNAs.

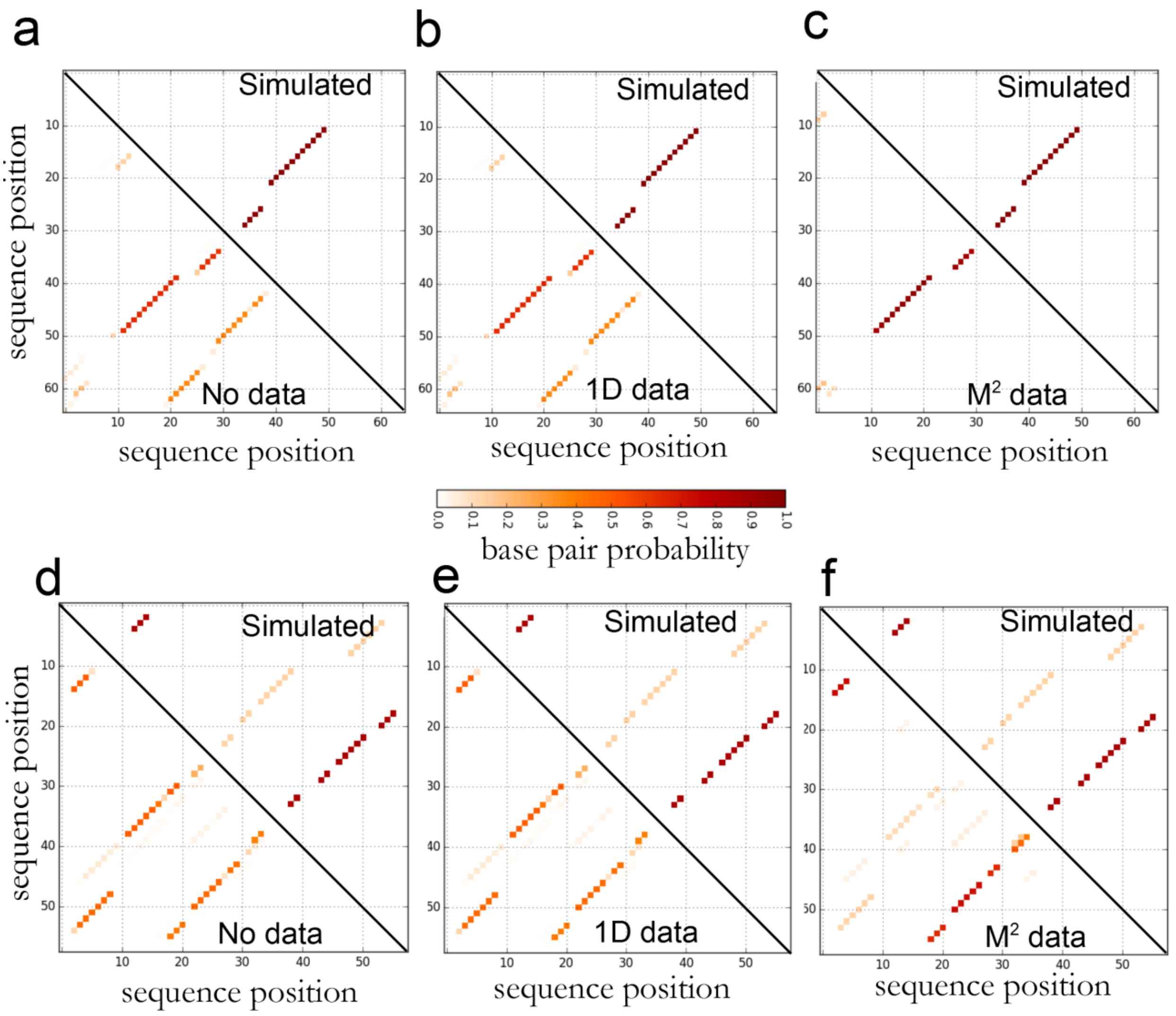

Figure E: Refinement of the 16S 126-235 RNA domain landscape model using mutate-and-rescue chemical mapping data. (a) Base pair probability matrix using only M^2 data. (b) Base pair probability matrix using M^2 and mutate-and-rescue data.

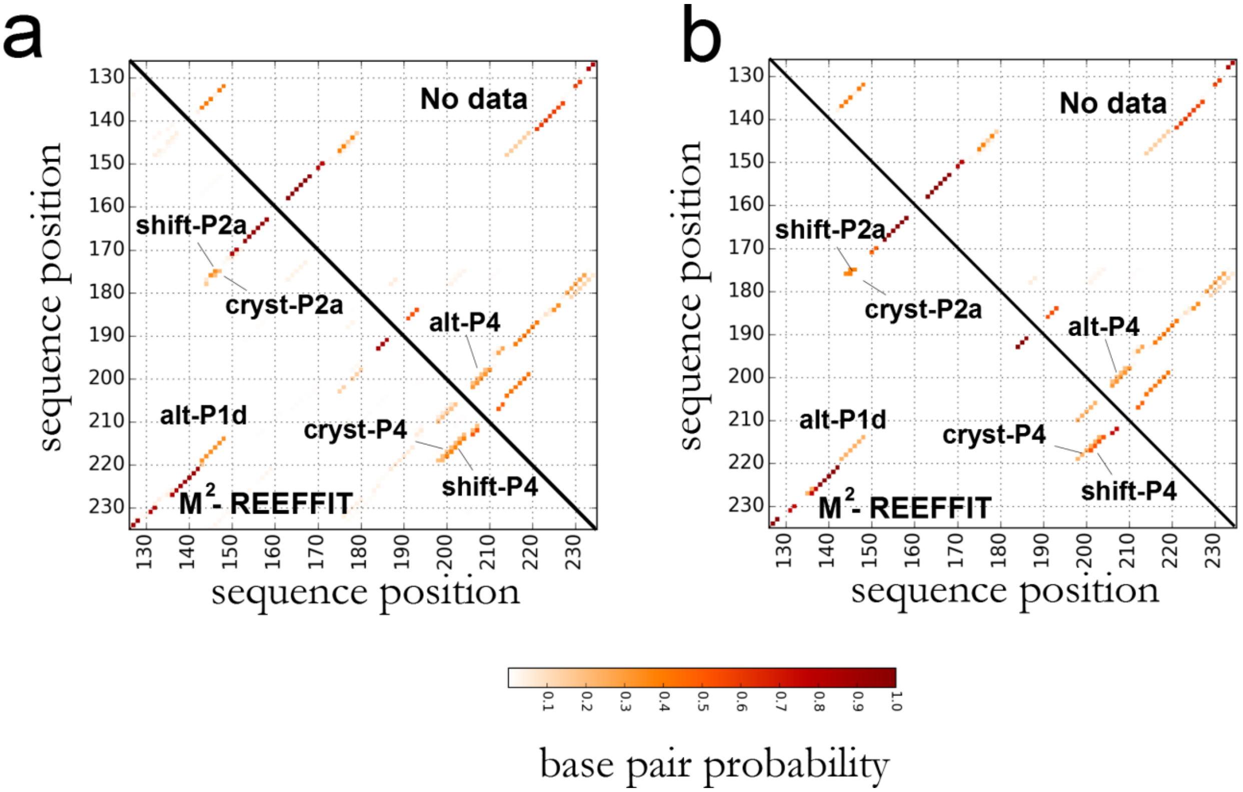

Figure F: Mutant-wise cluster weights for RNAs with previously-studied landscapes. (a) M^2 measurements of the MedLoop RNA (b) Mutant-wise weights for each cluster of structures, (c) representative state medioids. Analogous plots are shown for (d-f) the 16S rRNA 126-235 domain, (e-g) a bistable hairpin, and (h-k) the *add* adenine riboswitch. Cluster medioids are those shown in main text, Figs 2 and 3.

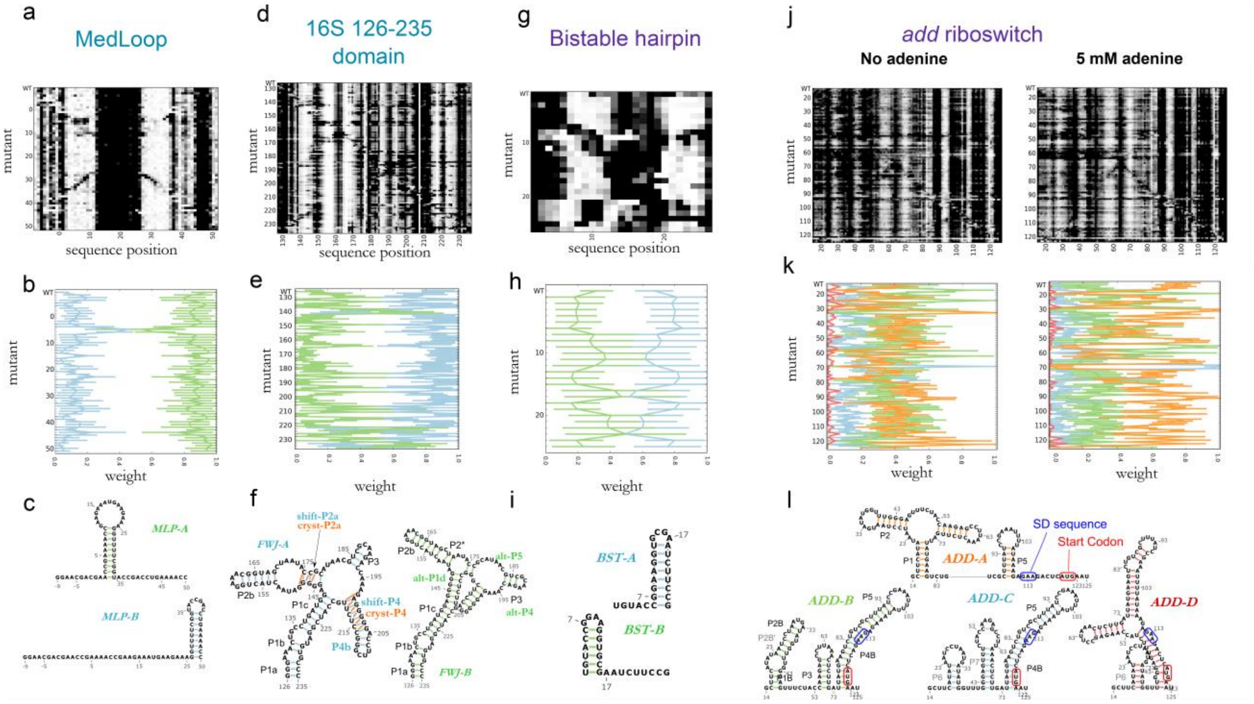

Figure G: Comparison of REEFFIT's global ensemble fit with a two-state fit on the Tebowned FMN.(a) M^2 data with no FMN; (b) predicted data using a global ensemble fit; (c) two-state fit using TBWN-A and TBWN-B. The two-state fit does not capture all the features seen in the experimental data. For example, mutants A25U and A34U are not fitted correctly in the two-state fit but are adequately captured in the global ensemble fit.

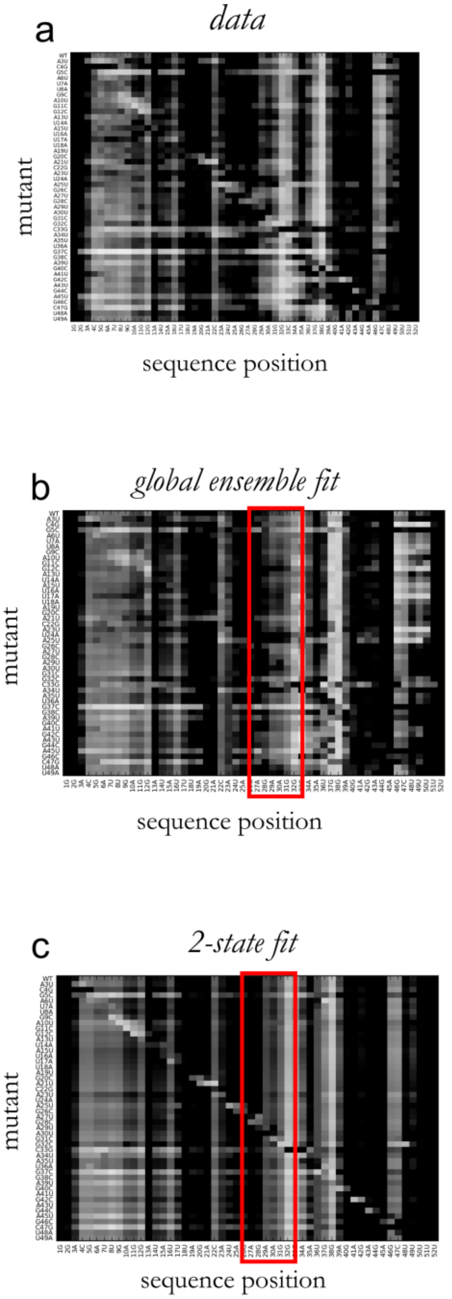

Figure H: Mutant-wise cluster weights for (a-c) the Tebowned switch and (d-f) the random M-stable RNA. Cluster medioids are those shown in main text, Fig.4 and 6, respectively.

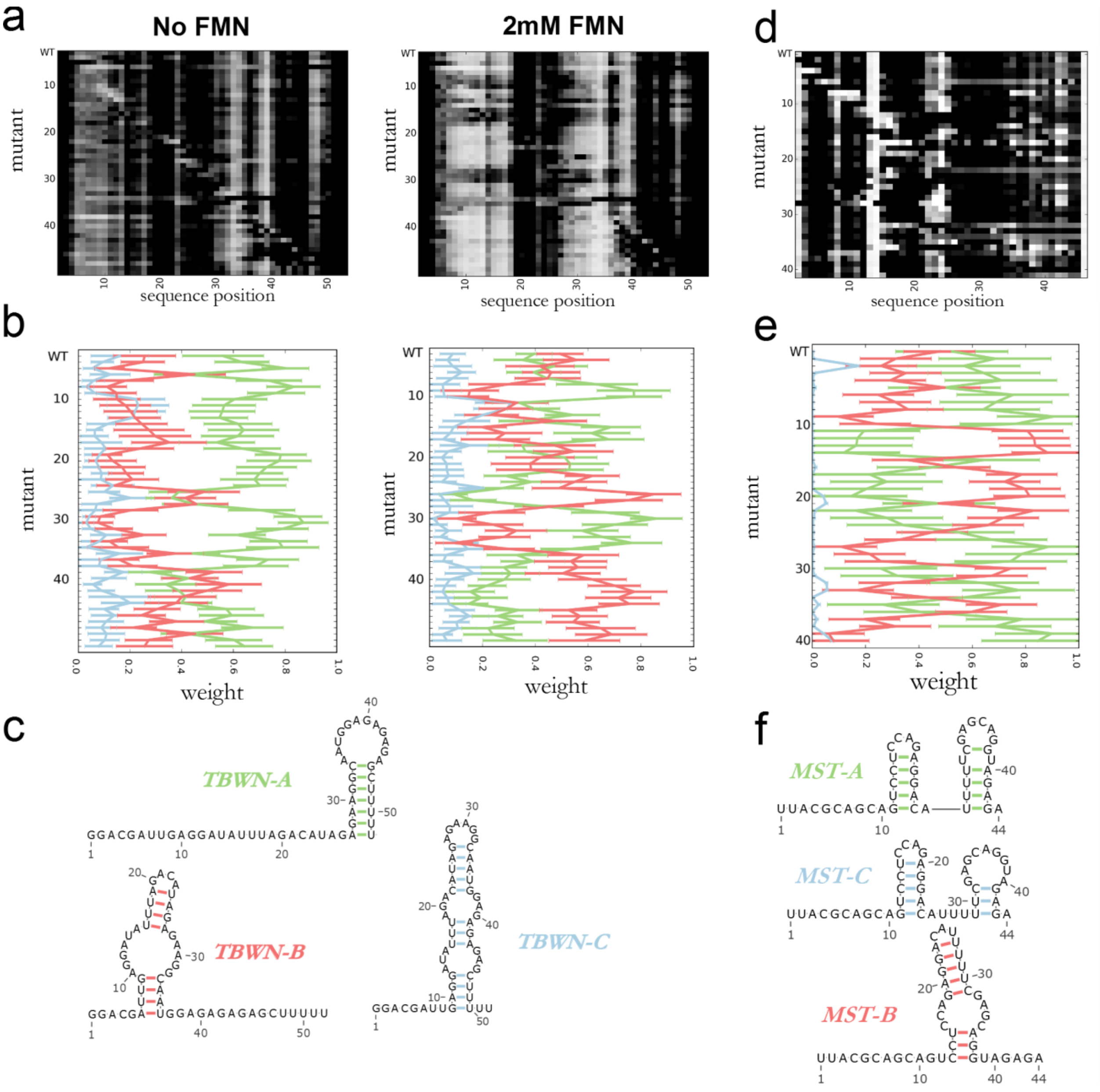

Figure I: DMS FMN titrations for each mutant stabilizing the TBWN-A (M1-A and M2-A), TBWN-B (M1-B and M1-B), and TBWN-C (M1-C and M2-C) structures used for the K_d fits in Figure 5.

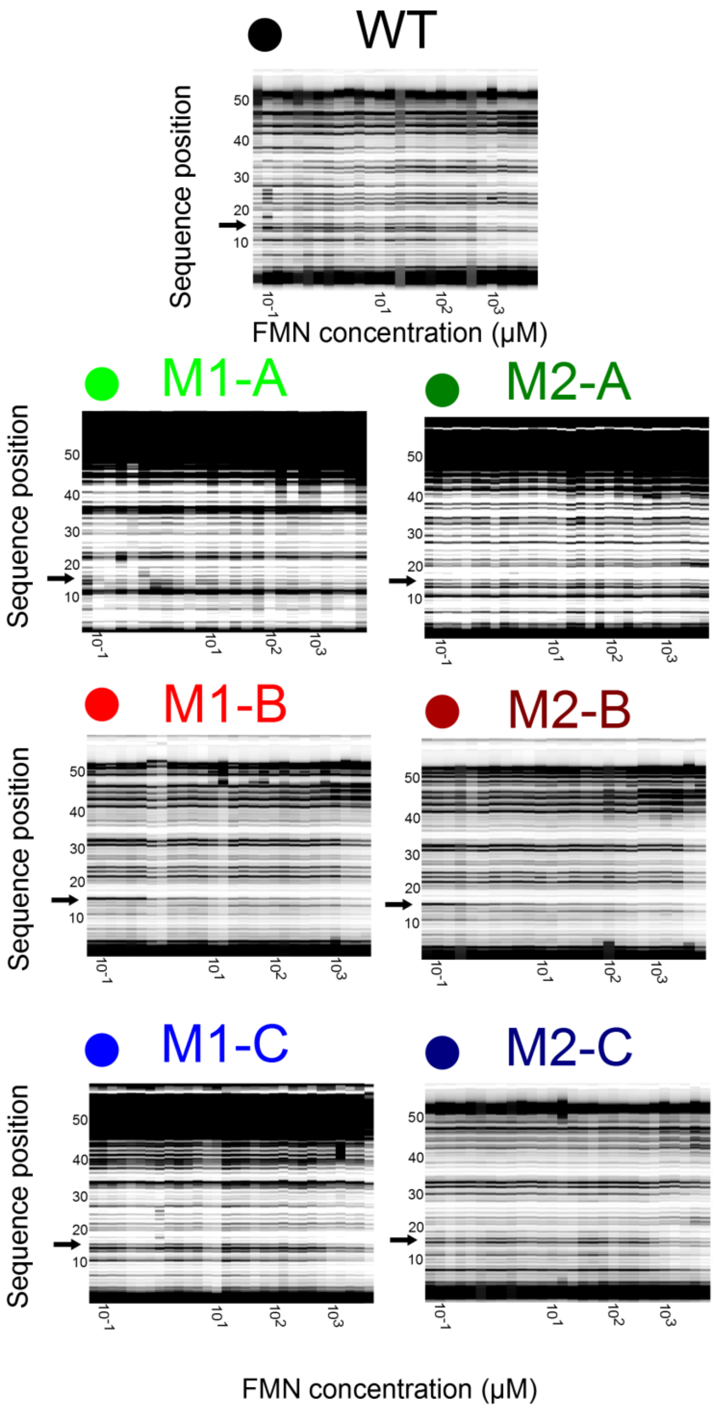

Figure J: Comparison of REEFFIT's global ensemble fit with two and three state fits for the M-stable random RNA. (a) M^2 data; (b) predicted data using a global ensemble fit; (c) 2-state, and 3-state fits using top structures as scored by RNAstructure free energy. Two and three state fits do not capture important features in the data (marked by blue arrows in (b)), such as patterns of protection in residues 14 to 45 found in several mutants (G5C, G21C, C31G, A33U, and A36U). (e.g. features of the C40G are not adequately captured in the 3-state fit but are adequately predicted by the global ensemble fit).

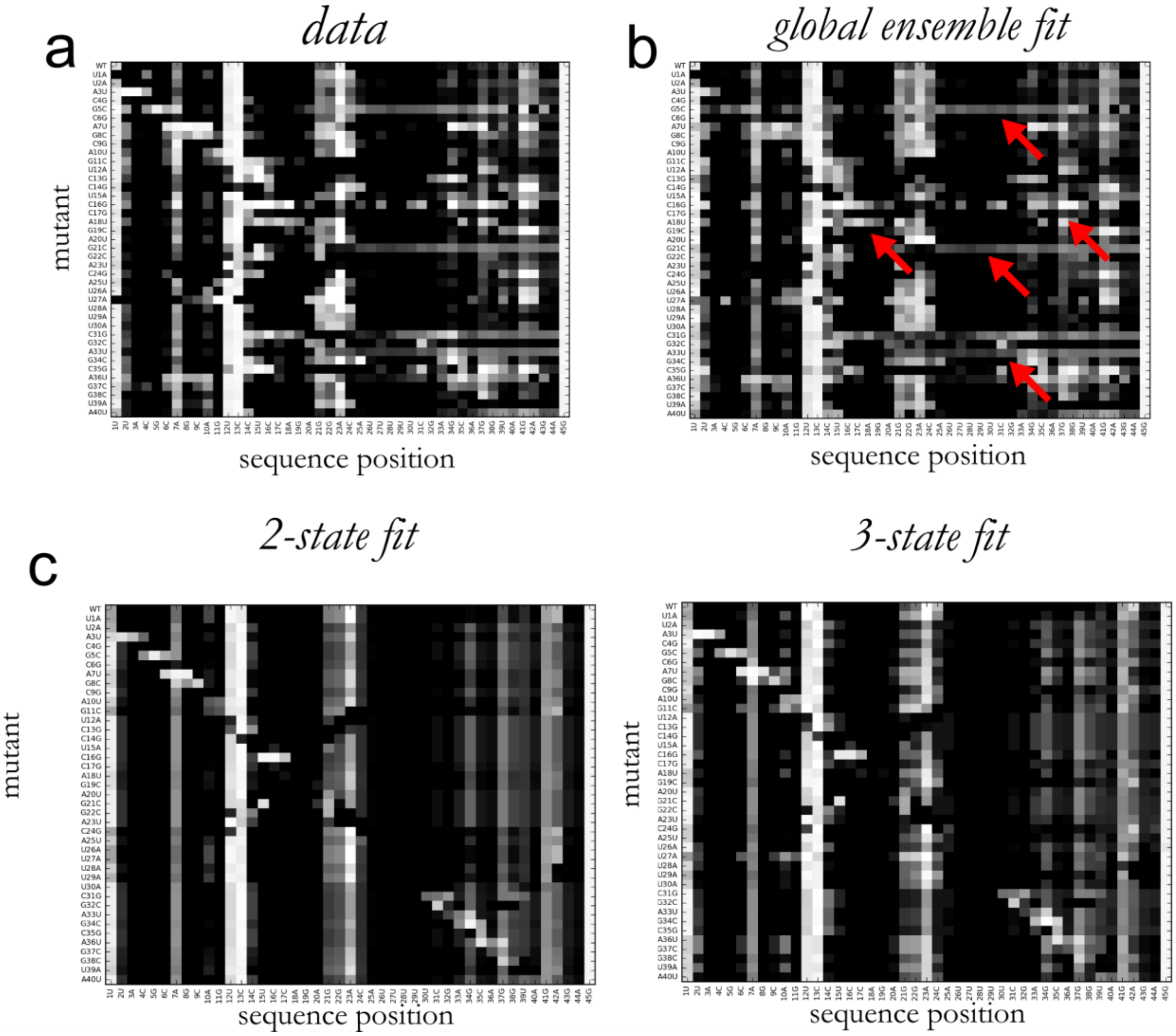

Supplement: S1 Text — (PDF) [file pcbi.1004473.s001.pdf]
